# Phase Coherent Currents Underlying Neocortical Seizure-Like State Transitions

**DOI:** 10.1523/ENEURO.0426-18.2019

**Published:** 2019-03-22

**Authors:** Vanessa Breton, Berj Bardakjian, Peter Carlen

**Affiliations:** 1 Department of Physiology, Faculty of Medicine, University of Toronto, Toronto, Ontario, Canada M5S 1A8; 2 Institute of Biomaterials and Biomedical Engineering, University of Toronto, Toronto, Ontario, Canada M5S 3G9; 3 Krembil Research Institute, Division of Fundamental Neurobiology, Toronto Western Hospital, Toronto, Ontario, Canada M5T 0S8; 4 Edward S. Rogers Sr. Department of Electrical and Computer Engineering, University of Toronto, Toronto, Ontario, Canada M5S 3G4; 5 Department of Medicine (Neurology), University Health Network, Toronto, Ontario, Canada M5G 2C4

**Keywords:** classification, electrophysiology, inhibition, prediction, seizure-like event, epilepsy

## Abstract

In the epileptic brain, phase amplitude cross-frequency coupling (CFC) features have been used to objectively classify seizure-related states, and the inter-seizure state has been demonstrated as being random, in contrast to the seizure state being predictable; however, the excitatory and inhibitory networks underlying their dynamics remain unclear. Therefore, the objectives of this study are to classify the dynamics of seizure sub-states labeling seizure-like event (SLE) onset and termination intervals using CFC features and to obtain their underlying excitatory/inhibitory cellular correlates. SLEs were induced in mouse neocortical brain slices using a low-magnesium perfusate, and were recorded in Layer II/III using simultaneous local field potential (LFP) and whole-cell voltage clamp electrodes. Classification of onset and termination of SLE transitions was investigated using CFC features in conjunction with an unsupervised two-state hidden Markov model (HMM). γ-Distributions of their durations indicated that both are predictable. Furthermore, omitting 4 Hz from the HMM classifier switched both SLE sub-states from statistically deterministic to random without changing the dynamics of the SLE state. These results were generalized to 4-aminopyridine (4-AP)-induced SLEs and human seizure traces. Only during these sub-states, both excitatory and inhibitory currents coupled with the field. Where excitatory currents phase locked to a broad range of frequencies between 1 and 12 Hz, inhibitory currents dominantly phase locked at 4 Hz. We conclude that inhibition underlies the predictability of neocortical CFC-defined SLE transition sub-states.

## Significance Statement

To date, the underlying excitatory and inhibitory bases of objectively identified seizure sub-states have not been determined. Here, we identify these sub-states using phase amplitude cross-frequency coupling (CFC) features. This is tested in both human and rodent seizure models. Then, using simultaneous field and whole-cell recordings in a mouse model, we investigate the potential for explaining seizure sub-state dynamics from interactions of the excitatory and inhibitory currents with local field network activity. We found that the frequency ranges at which these currents are coherent with field oscillations have a dramatic effect on the predictability of seizure state sub-states. Such information is critical for identifying the mechanisms underlying the dynamics of epileptic seizures.

## Introduction

Intractable epilepsy patients make up 30% of clinical cases, necessitating further investigation into potential treatment options (Kwan and Brodie, 2000). In studies of the outcomes from surgical resection for these intractable epilepsy patients, seizure freedom after surgery was markedly lower for neocortical epilepsy patients as compared to those whose seizures originated from the temporal lobe (Spencer and Huh, 2008; de Tisi et al., 2011). Yet, neocortical circuitry is not examined as widely as temporal lobe structures, which could contribute to why there are fewer successful targeted therapies for these patients.

An epileptic seizure is a period of complex electrical brain signals characterized by excessive pathologic neuronal activity (Fisher et al., 2014). Previously, the waveforms of high- and low-frequency oscillations (HFOs and LFOs) were examined independently as biomarkers of the seizure-prone brain (Ren et al., 2011; Jacobs et al., 2012); however, the pathologic rhythms were not separable from the physiologic. Yet, this separation is crucial for identifying underlying local networks involved in the pathology. Phase amplitude cross-frequency coupling (CFC) between LFO and HFO is a marker of neuronal excitability and communication (Lakatos et al., 2005; Mormann et al., 2005; Cohen et al., 2009; Canolty and Knight, 2010; Tort et al., 2010). Yet in the epileptic brain, it has been found that the ranges of coupled frequencies are different (Nariai et al., 2011; Guirgis et al., 2013, 2015; Samiee et al., 2018). There is, in particular, a marked increase in δ (0.5–4 Hz)-HFO CFC, which has been used to successfully identify resection zones for surgery and classify the seizure state (Ibrahim et al., 2013; Guirgis et al., 2013, 2014).

The seizure state is composed of multiple sub-states that include both the onset and termination transitions, but the underlying network processes that govern these sub-states remain unclear. Based on γ-distribution fits of interseizure and seizure duration histograms, seizure onset has been projected as randomly occurring (Suffczynski et al., 2006), whereas seizure termination has been demonstrated to be predictable (Bauer et al., 2017). However, there may be features that govern seizure sub-states that have predictable underlying patterns for both the onset and termination of the ictal events (Jacobs et al., 2018). In particular, using CFC to classify the ictal state may be more accurate and provide greater insight into state dynamics than visual inspection or amplitude-based measurements. Therefore, one of the main aims of this study was to identify the underlying dynamics of seizure sub-states using CFC features in the epileptic cortex.

A second aim of this study was to identify the underlying excitatory and inhibitory contributions to the CFC-identified seizure sub-states. During the onset of neocortical seizure-like events (SLEs), there is a large burst of inhibition synchronized with excitation (Kawaguchi, 2001; Timofeev and Steriade, 2004; Trevelyan et al., 2006; Lévesque et al., 2016; Librizzi et al., 2017; Chang et al., 2018). Furthermore, at termination, there may be a weak recovery of inhibitory currents, with a concomitant slowing down of large excitatory field bursts (Köhling, 2014). Yet, it is unknown how these excitatory and inhibitory patterns relate to the dynamics of the seizure sub-state durations. Furthermore, although combinations of LFO and HFO have been previously used to classify multiple seizure state sub-states (Guirgis et al., 2014), only extracellular recording techniques were applied. Hence, their underlying excitatory and inhibitory currents were not investigated. Therefore, simultaneous extracellular and intracellular measurements provide a substantial advancement in identifying the underlying cellular mechanisms of seizure sub-states.

In brief, our first aim is to identify the relation between CFC-identified seizure sub-states and the predictability of these classified intervals, and our second aim is to investigate the excitatory and inhibitory currents associated with these CFC features. To achieve this, simultaneous measurements of extracellular local field potential (LFP) and whole-cell recordings were made in the superficial layers of the mouse somatosensory cortex. A novel CFC based feature set was applied to a two-state hidden Markov model (HMM), and was used to classify neocortical SLE states, with onset and termination sub-states indicated by the marginal errors in the HMM. This was tested in low Mg^2+^, and 4-aminopyridine (4-AP), and then the results were generalized to human intracranial EEG (iEEG) signals. Finally, the excitatory and inhibitory current patterns underlying the different HMM-identified SLE state transitions were determined.

## Materials and Methods

### Animals

C57/Bl6 (RRID:IMSR_JAX:000664) male and female mice aged P10 to P16 (Charles River Laboratories, RRID:SCR_003792) were humanely killed for these experiments in accordance with the Canadian Animal Care Guidelines and with the United States Public Health Service’s Policy on Humane Care and Use of Laboratory Animals. All surgical procedures were approved and done in accordance with the guidelines of the Animal Care Committee of the University Health Network.

### Cortical slice preparation

To prepare the cortical tissue for resection, mice were anesthetized using 50-mg/kg pentobarbital. The pedal reflex was used to assess the depth of anesthesia. Once deeply anaesthetized, the mice were decapitated, and then their whole brain was swiftly removed. The brain was placed in an ice cold, oxygenated sucrose solution made up of 248 mM sucrose, 2 mM KCl, 3 mM MgSO_4_, 1 mM CaCl_2_, 26 mM NaHCO_3_, 1.25 mM NaH_2_PO_4_, and 10 mM D-glucose (Florez et al., 2015). Then, to limit the contribution of distal projections to induced epileptiform activity, the lateral 20% of both cerebral cortices was excised, and the remainder of the cortex was fixed to a vertical block using a cyanoacrylate adhesive. Coronal slices (500 µm) were sectioned using a Leica 1200 V vibratome. Then, each slice was hemisectioned and transferred to a solution of artificial CSF (ACSF; 95% O_2_, 5% CO_2_) containing 123 mM NaCl, 25 mM NaHCO_3_, 10 mM glucose, 3.5 mM KCl, 1 mM MgSO_4_, 1.2 mM NaH_2_PO_4_, and 1.5 mM CaCl_2_. The slices were incubated for 30 min at 32 ± 0.5°C followed by 1 h at room temperature before the start of the experiments.

### Electrophysiology

The slices were transferred individually to a submerged recording chamber and were perfused with ACSF (95% O_2_, 5% CO_2_; 10 ± 1 ml/min; 34 ± 0.5°C). Glass electrodes (1.5 mm, World Precision Instruments) were used for both LFP and whole-cell recordings. The LFP recording electrodes (∼2-MOhm resistance) contained ACSF and were placed within 200 µm of the intracellular electrodes in cortical Layers II and III. An Olympus BX51 microscope (OLY-150IR camera–video monitor unit) was used with an infrared filter to visualize the neurons. The whole-cell recording electrodes (3–5 MOhm resistance) contained a solution of 135 mM K-gluconate, 1 mM MgCl_2_, 10 mM NaCl, 2 mM Na_2_ATP, 0.3 mM NaGTP-Tris, 10 mM NaHEPES, 0.5 mM EGTA, and 0.0001 mM CaCl_2_; pH 7.2–7.3. Signal acquisition and storage were performed using an amplifier (Multiclamp 700B), a digitizer (Digidata 1322A) and PClamp software (version 10.2; Molecular Devices/Molecular Devices Corporation). Whole-cell recordings were done in voltage clamp. Neurons were held at –70 mV for spontaneous EPSC (sEPSC) acquisition and 0 mV for spontaneous IPSC (sIPSC) acquisition. In 3/6 experiments for sIPSCs, CsCl replaced K-gluconate to prevent potassium current contamination of the intracellular signal. In these cases, putative pyramidal neurons were solely identified based on their appearance under the infrared filter. Furthermore, the addition of the CsCl did not affect the temporal correlation between the inhibitory currents and field activity; therefore, all inhibitory current data were combined for signal analysis.

Putative pyramidal neurons were identified based on their appearance under the infrared filter and their electrophysiological features. A series of hyperpolarizing and depolarizing current pulses (200-ms duration) were injected through the whole-cell recording electrode. We computed the following to confirm putative pyramidal neuron identity: an average input resistance of 150.75 ± 10.63 MΩ (*n* = 16 cells) was calculated from the slope of the current−voltage curve. A spike half-width of 1.59 ± 0.07 ms was obtained from the first spike at rheobase, and defined as the duration of the action potential at the membrane voltage 50% from threshold to peak. Finally, a membrane time constant of 19.61 ± 1.41 ms was obtained from fitting an exponential to the first 50 ms of the minimally injected current. These values reported are consistent with those reported in the literature (Avermann et al., 2012; van der Velden et al., 2012; Tyler et al., 2015). However, we did not correct for liquid junction potential since all results central to the hypothesis were measured against a change from baseline in voltage clamp. To assure only healthy neurons were used, only those whose resting membrane potential before SLE induction greater than –65 mV were studied; furthermore, any recording with a change in the input resistance >20% during the inter-SLE phase relative to the ACSF baseline was omitted from further analysis.

Tissue viability was assessed in two ways. First, we observed that all slices exhibited greater than one SLE. Second, SLE amplitude did not decrease over time. Therefore, only tissue with sustained intrinsic seizure control mechanisms and good slice viability was used for this study (Voss et al., 2013). For SLE induction, MgSO_4_ was omitted during the preparation of the ACSF (low Mg^2+^), or 4-AP (100 µM) was applied to ACSF containing normal levels of MgSO_4_ (Wang et al., 2016).

### Patient description

Nineteen seizures recorded from iEEG grids placed on the neocortex were obtained from seven patients with intractable epilepsy. Seizures were obtained from patients at Toronto Western Hospital (Toronto, Canada) and Phramongkutklao Hospital (Bangkok, Thailand). Data from these patients were used in previous studies examining HFO coherence, CFC in seizure onset zone classification and seizure predictability (Cotic et al., 2015; Guirgis et al., 2015; Li et al., 2016; Jacobs et al., 2018). Patients and the ethics committees of the affiliated institutions provided informed consent and approval. All patients were seizure free (Engel class 1) after surgery. For each seizure, a bipolar recording from an 8 × 8 iEEG multi-electrode recording taken from electrodes nearby the resected zone was chosen for the analysis. This onset zone had been previously defined by two expert neurologists. These recordings were acquired at sampling frequencies of 500, 1000, and 2000 Hz. The data obtained from Canada was notch-filtered at 60 Hz and its harmonics up to Nyquist frequency, and the data obtained from Thailand were notch-filtered at 50 Hz and its harmonics up to Nyquist frequency. Before feature extraction, all signals were decimated to 500 Hz.

### Data analysis

#### CFC-based feature set

For an objective way to classify the SLE state and SLE sub-states, a novel CFC feature set was applied to a two-state HMM. First, all LFP recordings were decimated to 1000-Hz sampling rate then FIR notch filtered at 60 Hz and its first five harmonics. The instantaneous phase of the LFOs (1–12 Hz) and amplitude of the HFOs (30–250 Hz), were computed by convolution of the filtered LFP signal with a complex Morlet wavelet as defined by:

(1) $$S\left(t,a\right)=\int_{-inf}^{inf}x(\tau )\frac{1}{\sqrt{a}}W\left(\frac{\tau -t}{a}\right)d\tau$$

(2) $$W\left(t;f_{b},f_{c}\right)=\frac{1}{\sqrt{\pi f_{b}}}e^{2i\pi f_{c}t}e^{-}{}^{t^{2}/f_{b}}$$

**Figure 1.**
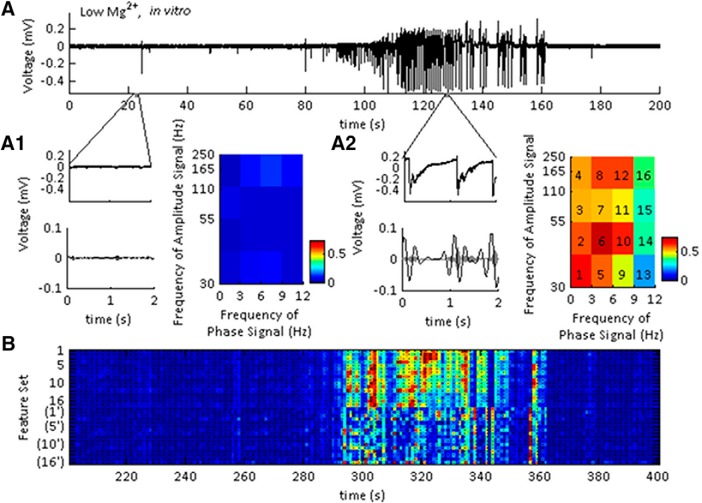
Building the feature set for SLE state classification using I_CFC_ matrices. ***A***, LFP recording from the neocortex showing a representative SLE induced in low-Mg^2+^ ACSF. Regions expanded for comparison are highlighted by gray bars. ***A1***, Expanded pre-SLE region unfiltered (upper), and filtered (lower). The lower trace was band pass filtered at 3–6 and 30–40 Hz, indicated by the black and superimposed gray lines, respectively. This range was chosen to represent the frequency space shown to have highest I_CFC_ values in that instance. The right image is the I_CFC_ matrix of a 2-s window including the expanded region, demonstrating low I_CFC_ over the full frequency spectrum, with peaks at 3–6 and 30–40 Hz. ***A2***, Expanded region of the SLE state unfiltered (upper), and filtered (lower). The lower trace was band pass filtered at 1–3 and 30–40 Hz, indicated by the black and superimposed gray lines, respectively. This range was chosen to represent the frequency space shown to have highest I_CFC_ values in that instance. The right image is the I_CFC_ matrix of a 2-s window including the expanded region, demonstrating peak I_CFC_ over a small range of the frequency spectrum. The numbers on the image indicate the features unwrapped to make the feature set. ***B***, The feature set obtained from the signal in ***A*** used for classifying the SLE state. The numbers on the vertical axis correspond to the frequency ranges indicated by the numbers on the pseudocolor plot in ***A2*** (1–16) and their time derivatives [(1’)–(16’)]. Each row was normalized to the maximum and minimum I_CFC_ values across the full trace.

These were computed at f_b_ = 3-Hz bandwidth, with f_c_ = 1-Hz center frequency. To avoid edge effects, a 5-s window on either side of the signal of interest was kept during the computation of the continuous wavelet transform (cwt), and then subsequently removed for calculation of the CFC indices (I_CFC_). Then the I_CFC_ was estimated on 2-s sliding windows. For every 2-s window, the instantaneous phase signal was divided into 20° equal intervals. In each phase interval, the amplitude, *A_high_*, was averaged then normalized by the sum of the average amplitudes (Tort et al., 2010):

(3) $$A_{norm}(j)=\frac{{\left(A_{high}\right)}_{j}}{\sum_{k=1}^{N}{\left(A_{high}\right)}_{k}}$$

where *N* is the number of phase intervals and j is a discrete phase interval. The Kullback–Leibler (KL) distance was computed between the A_norm_ and a uniform distribution. Then, the KL distance was normalized to an interval between 0 and 1. This normalized KL distance was the phase amplitude CFC index (I_cfc_; also known as modulation index) for each data point on the CFC image. Then, the I_CFC_s used for training and testing the HMM were obtained from the I_CFC_ matrix on first modifying the vertical axis to be logarithmic, then re-shaping the image to a 4 × 4 matrix (Fig. 1; features 1–16). The remaining features were the derivatives of the initial set of indices [Fig. 1*B*, features (1’)–(16’)]. The features used for training were obtained using 2-s windows for the I_CFC_ computation and 0% overlap on the sliding window function. The features used for testing were obtained similarly, using 2-s windows and 95% overlap.

#### I_CFC_ control analysis

To identify significant ranges of CFC, surrogate data were generated by converting the original time series into a complex series using the cwt (1- to 12-Hz intervals in 1-Hz increments). The phase was then random block-point shuffled with blocks of 1% of the sampling frequency (10 data points, randblock.m). Then the CFC indices were computed on 5-s windows at the classified r_o_ onset of the SLEs and classified r_e_ end of the SLEs. The amplitude coefficients of the high-frequency cwt (30–250 Hz, in 5-Hz increments) were used, with the surrogates, to compute the CFC indices. This was repeated 200 times. The original CFC was compared to the surrogate data either at onset or termination. The CFC was *z* score normalized to the mean and standard deviation of the distribution of the surrogate values. Ranges considered significant were those whose distributions were above 3× the SEM of the surrogate dataset. This test for significance provided the boundaries of significant CFC for further control analysis.

#### HMM state classification

We used a first order Q = two-state HMM which, for our application, delineated SLE and inter-SLE states S1 and S2 at a time t, where 1 ≤ t ≤ T, and T indicates the length of the feature set (Guirgis et al., 2014). The HMM requires estimating the following distributions {*s,* ***A, B***}: for i = 1,2 and *j =* 1,2 (1) *s = Pr(q_1_ = j)*; the probability of initially being in state *Q = q_j_.* (2) ***A_t_****= Pr(q_t_ = j* | *q_t-1_= i);* the probability of transitioning from one state to another at time *t*. (3) ***B_t_*** *= Pr(X_t_ = x* | *q_t_ = j) = {b_j_(****O****)};* the probability of observing *x,* a given value of the feature set, when in the state *q* at a given time. ***O*** is a 2-dimensional set of observed features for state j. The model values for {*s,* ***A, B***} were initially estimated using the *k*-means clustering algorithm, and then their accuracy was enhanced using the expectation-maximization (EM) algorithm. To compute ***B,*** each {***O****}* set of features was fit using a mixture of Gaussians with the probability density function (PDF) defined as

(4) $$PDF\left(\Delta ,\mu_{m},CV_{m}\right)=\frac{exp\left(-0.5{\left(O-\mu_{m}\right)}^{T}CV_{m}^{-1}\left(O-\mu_{m}\right)\right)}{{\left(2\pi \right)}^{\frac{K}{2}}){\left|CV\right|}^{0.5}}$$

where µ_m_ is the mean, CV_m_ the covariance and *m = 1 or 2* is the number of Gaussian basis functions. ***B* =** *{b_j_(****O****)}* is the sum of the weighted Gaussian probability densities over all the basis functions.

The marginal posterior distributions (MPDs) of the HMM were computed as follows:

(5) $$MPD(i)=\frac{\alpha_{t}(i)\beta_{t}(i)}{\sum_{i}\alpha_{t}(i)\beta_{t}(i))}$$

(6) $$\alpha_{t}(i)=\left(\sum \alpha_{t-1}\alpha_{ij}\right)b_{i}\left(O_{t}\right)$$

(7) $$\beta_{t}(i)=\sum a_{ij}b_{j}\left(O_{t+1}\right)\beta_{t+1}(i)$$

where α is the joint probability of observing all data from time 1:t; and β is the conditional probability of all data from t + 1 to T at state i. Then, the log likelihood evaluated the goodness-of-fit between the feature set of training observations ***O,*** and the model parameters {*s,* ***A, B***}. The process was repeated to the point where either the difference between log likelihood values of successive iterations dropped below 10^−5^ or 100 iterations took place. Generally, the log likelihood converged within 11–25 iterations and in no case did it reach 100 iterations. The stem plots are the *MPD(1)* and *MPD(2)* in testing conditions, which was re-named as S1 and S2 to represent the inter-SLE state and SLE state, respectively.

A γ-distribution parameterization of time histograms was performed using the gamfit function in MATLAB. The γ-distribution is characterized by a shape and a rate parameter. The maximum likelihood estimates of the shape parameter, α, led to the following inferences of the dynamics of the SLE state intervals (Bauer et al., 2017):

(5) $$\alpha =\left\{ \begin{array}{l} <1\;stochastic\\ =1\;Poisson\\ >1\;deter\;ministic \end{array}\right.$$

**Figure 2.**
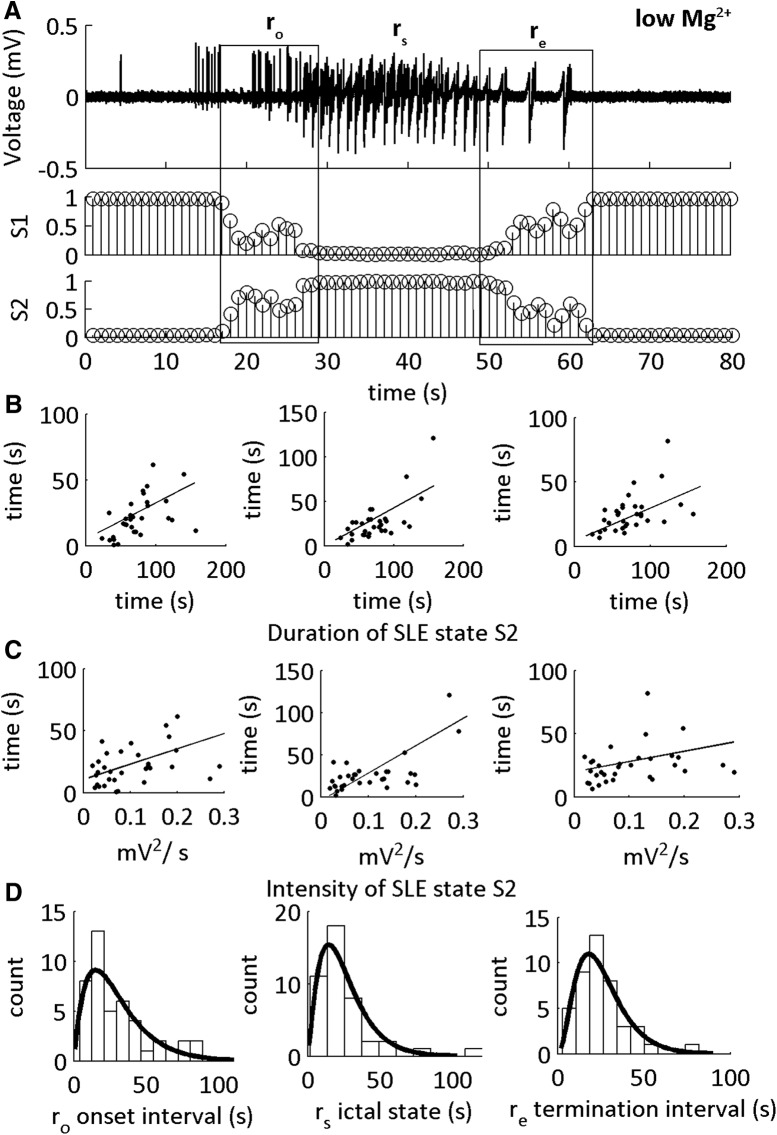
I_CFC_ features classify low-Mg^2+^-induced SLE sub-states. ***A***, LFP recording from neocortical Layer II/III showing a representative SLE induced in low-Mg^2+^ ACSF, and the MPDs S1 and S2. Boxes indicate putative SLE transitions at onset (r_o_) and end (r_e_) of the SLE state (r_s_). These time windows are obtained from 0 → 1 and 1 → 0 fluctuations of the MPDs S1 and S2. ***B***, Scatter plots showing a moderate positive correlation between the duration of r_o_, r_s_, and r_e_ and the duration of the full SLE state. Each data point is one SLE. ***C***, Scatter plots showing a moderate positive correlation between state r_s_ and the r_o_ transition, and the intensity of the SLE state and weak correlation between the transitions r_e_ and the intensity of the SLE state. For Pearson correlation coefficients, see Table 1, lines 1–6. ***D***, Histograms of the durations of r_o_, r_s_, and r_e_ with their respective γ-distribution fits. See Table 2 for shape parameters of the γ-distribution fits and average durations of each interval. All distributions fit to a shape parameter >1.

The SLE state S2 was subdivided into putative SLE transitions within state S2 at onset (r_o_) and end (r_e_) of the SLE state (r_s_). These time windows were obtained from 0 → 1 and 1 → 0 fluctuations of the MPD S2. Duration and intensity of the SLE state S2 was computed (Fig. 2*B*,*C*) using the following operations: *Duration(S2) = L(r_o_) + L(r_s_) + L(r_e_)*; *Intensity(S2) = (V_L_)^2^/L*; where V_L_ is the LFP signal within a time interval, *L,* of the sub-sections of S2 (r_o_, r_s_, r_e_). A Pearson correlation coefficient was applied to measure the linear correlation between the duration of the sub-intervals of the SLE state S2 and that of the duration and intensity of the SLE state S2 (0 → no correlation, 0.1–0.3 → weak correlation, 0.31–0.69 → moderate correlation, 0.7–1 → strong correlation).

**Figure 3.**
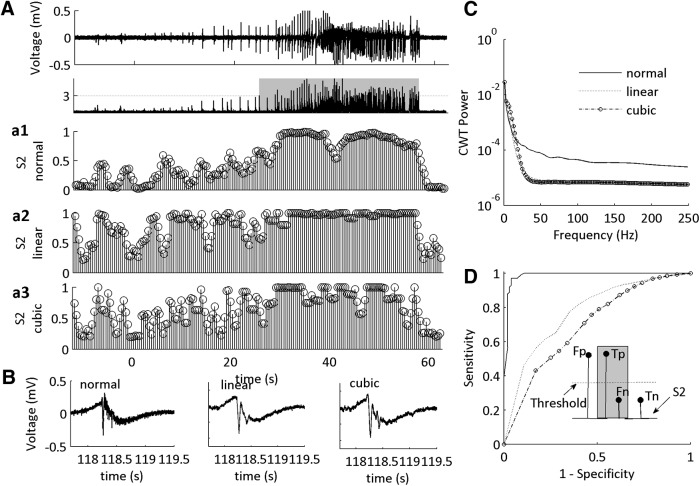
Reconstructed data containing low-frequency activity from low-Mg^2+^ SLEs poorly classifies the SLE state. ***A***, Example of LFP trace of a low-Mg^2+^ SLE (top). The signal power is used as an alternate definition of the SLE state (bottom) and is applied to the computation of ROC curve. Gray box indicates defined SLE state. ***a1***, MPD of S2 of the LFP presented in ***A***. ***a2***, MPD of linear interpolated reconstructed data obtained from the envelope of the SLE in ***A***. ***a3***, MPD of cubic interpolated reconstructed data obtained from the envelope of the SLE in ***A***. ***B***, One example of a single burst from the SLE in ***A*** at 118 s under normal conditions (the unfiltered dataset), using a linear interpolant (reconstructed data) and using a cubic interpolant (reconstructed data). ***C***, Example of the power spectra of the three datasets. The low-frequency power remains similar across models, but the high-frequency power is minimized. ***D***, ROC curves for the three conditions for all SLEs tested. For a summary of the ROC characteristics, see Table 4 (*n* = 46 reconstructed SLEs, 14 slices, 10 subjects). Inset is a representation of the computation of the ROC sensitivity and specificity. The square wave shows a typical MPD of the HMM. The lines are depictions of the stem plots of the MPD of state S2. Fp is false positive, Tp is true positive, Fn is false negative, Tn is true negative.

Performance of the HMM classifier was determined using receiver operator characteristic (ROC) curves. To compare to a standard approach in defining seizure onset and termination, these were identified using the LFP power. The LFP power was computed by squaring the absolute value of the cwt and averaging over the full SLE state. The SLE onset was defined as the time where the LFP signal rose to 3× the baseline signal. The SLE termination was defined as a return to baseline after a final field bursting event, followed by >10-s refractory period. If the area-under the curve (AUC) is 1, that is a perfect classification, whereas if it is 0.5 or less, it is a worthless classification (for an example, see Fig. 3*A*). During the inter-SLE interval, false positive (Fp) was obtained if, for each point on the MPD of state S2, state S2 was classified as higher probability than state S1, and the opposite was considered true negative (Tn). During the SLE interval, true positive (Tp) was when S2 remained in higher probability than S1, and if the probability distribution switched, this was false negative (Fn). The threshold for defining the SLE interval was varied between 0 and 1 on S2 to obtain the points on the ROC curve. Sensitivity, specificity and a specificity factor, ξ, were computed as follows:

(6) $$Sensitivity=\frac{\sum Tp}{\sum Tp+Fn}$$

(7) $$1-Specificity=\frac{\sum Fp}{\sum Fp+\sum Tn}$$

(8) $$Sensitivity=1-exp\frac{1-Specificity}{1-\xi }$$

Optimal sensitivity and specificity values were also reported from the specificity factor ξ-exponential curve fit. These were reported as the nearest point on the exponential function relative to the coordinate (0,1).

#### Controls for I_CFC_ classified using HMM

To account for the possibility that the irregular shape of the low-frequency signal alone is sufficient to capture the dynamics of the SLE state using CFC features, data with minimal HFO contribution was simulated by reconstructing the original datasets. We obtained the envelope function of the original LFP dataset (envelope.m), and then added Gaussian white noise (wgn.m) of the same variance as the original dataset computed in a 10-s window of quiescence during the pre-SLE state. To account for variability in the wave form shapes during SLEs, this was repeated using two variations of the envelope interpolation: linear and cubic.

To identify whether large spiking transients would affect the classified state transitions, data were first bandpass filtered 30–250 Hz using 10,000 order finite impulse response (FIR) filters that were run in the forward and reverse direction (filtfilt.m). Then, a spike melting algorithm (spikinator.m) was applied to smoothly remove all spikes above a specified threshold (Zalay et al., 2009) using the following parameters: 10-ms window, threshold of 1.2, amplitude of 0.01, smoothing of 1, degree of spike cleaning of 0.8, and window frame margin of 0.1. These parameters were chosen on inspection of the amplitude of the large spiking transients. This high-frequency amplitude signal was used in conjunction with the phase of the low-frequency 1- to 12-Hz oscillation and feature sets were built as previously defined. We only used the CFC features directly (features 1–16, no derivatives) for this test. Each new signal was tested on an HMM that was trained on its corresponding original signal. The % difference between the S2s was defined as the sum of the difference between the MPD of S2 of the original signal and the MPD of S2 of the spike reduced signal, divided by the total number of points tested.

#### Charge transfer energy of the cellular currents

To evaluate the charge transfer energy of the sEPSCs and sIPSCs, the trapezoidal rule was applied. Because each SLE sub-state was of different duration, data were evaluated in amortized time. The SLE sub-state duration was divided into 10 time intervals of equal duration, and the energy was averaged over each 10% of the 100% of sub-state duration. All data were statistically tested relative to the 10% before the onset transition sub-state, r_o_.

#### Peak phase coherence between the cellular currents and the LFPs

For identifying the correlation between the timing of the synaptic events and the phase of the LFP oscillation, first, the timing of spontaneous PSCs (sPSCs) was identified using an amplitude threshold and the findpeaks.m function in MATLAB, with a minimum peak distance of 50 ms and minimum peak height of 3× SD of the 10% pre-SLE area. Then, at each time of the sPSC peak, the phase at which the event occurred was computed as the instantaneous phase of the cwt, as previously defined. Phase vector plots include the phase distribution of the peak sPSCs, mean phase direction (bold black lines), mean phase vector length (number at top) and scale (relative probability value). The mean phase vector direction is the mean phase at which the peaks are occurring. The mean phase vector length is the degree of variability in the timing of the peaks around the mean; therefore, the higher the mean phase vector length, the stronger the phase locking. The mean phase vector length is the phase locking value (PLV) used in a recursive algorithm.

Using a recursive algorithm in MATLAB, we varied the threshold for peak identification and calculated the phase of the low-frequency signal (between 1 and 12 Hz) until we observed significant elevated phase locking during the SLE transition sub-state. For each phase vector plot, the Rayleigh test was used as a statistical test of circular uniformity (CircStats toolbox), with a *p* < 0.05 threshold for significance. The algorithm included a criterion to identify PLVs >0.3 (indicating moderate phase locking) and *p* < 0.05. For visualization purposes, the filtered version of the LFP at the frequency range of interest was plotted superimposed on a stem plot where the stems are located at each detected sPSC.

### Experimental design and statistical analysis

Statistical analyses were performed using MATLAB and Microsoft Excel. An unpaired *t* test was used when there were two groups and a normal distribution, Wilcoxon rank-sum test when there were two groups and non-normal distribution. A Bonferroni post-hoc correction was applied in each case for tests of multiple comparisons. Pearson Correlation was used for analyses of correlation between two variables. Details of numbers of SLEs, slices and subjects evaluated are reported in appropriate subsections of figure legends. The α threshold was set to 0.05 (for further details, see Table 1).

**Table 1.** Statistics table

Line number	Figure	Data structure	Test	CI of difference of mean/median bounds		*p* value	Adjusted *p* value	*r*
1	2*B* r_o_	Linear correlation	Pearson correlation coefficient			0.0025		0.52
2	2*B* r_s_	Linear correlation	Pearson correlation coefficient			7.80E-06		0.7
3	2*B* r_e_	Linear correlation	Pearson correlation coefficient			0.0026		0.51
4	2*C* r_o_	Linear correlation	Pearson correlation coefficient			0.032		0.38
5	2*C* r_s_	Linear correlation	Pearson correlation coefficient			6.40E-05		0.65
6	2*C* r_e_	Linear correlation	Pearson correlation coefficient			0.11		0.28
7	4*B*	Linear correlation	Pearson correlation coefficient			0.039		0.3788
8	8*C*_SLE1,lowMg_	Non-Gaussian		–2.604	3.774	Baseline		
9	8*C*_SLE2,lowMg_	Non-Gaussian	Wilcoxon rank-sum test, multiple comparisons using Bonferroni's correction	–0.976	2.344	0.1635	0.4905	
10	8*C*_SLE3, lowMg_	Non-Gaussian	Wilcoxon rank-sum test, multiple comparisons using Bonferroni's correction	–1.752	14	0.8114	0.99	
11	8*C*_SLE4,lowMg_	Non-Gaussian	Wilcoxon rank-sum test, multiple comparisons using Bonferroni's correction	-5.325	9.581	0.9319	0.99	
12	8*C*_SLE1,4-AP_	Non-Gaussian	Wilcoxon rank-sum test, multiple comparisons using Bonferroni's correction	7.2357	1.079	Baseline		
13	8*C*_SLE2,4-AP_	Normal (non-Gaussian baseline)	Wilcoxon rank-sum test, multiple comparisons using Bonferroni's correction	3.4356	–4.948	0.115	0.345	
14	8*C*_SLE3, 4-AP_	Non-Gaussian	Wilcoxon rank-sum test, multiple comparisons using Bonferroni's correction	0.4743	–8.075	0.0763	0.2289	
15	8*C*_SLE4, 4-AP_	Non-Gaussian	Wilcoxon rank-sum test, multiple comparisons using Bonferroni's correction	6.109	–1.429	0.0224	0.0672	
16	9_left (CFC significant) mean_	Linear correlation	Pearson correlation coefficient	0.20 ± 0.03				0.49 ± 0.04
17	9_left (CFC not significant) mean_	Linear correlation	Pearson correlation coefficient	0.12 ± 0.03				0.6 ± 0.04
18	9_right (CFC significant) mean_	Linear correlation	Pearson correlation coefficient	0.21 ± 0.05				0.43 ± 0.05
19	9_right (CFC not significant) mean_	Linear correlation	Pearson correlation coefficient	0.043 ± 0.02				0.59 ± 0.04
20	10*C* r_o point 1_	Non-Gaussian		0.8188	0.923	Baseline		
21	10*C* r_o point 2_	Non-Gaussian	Wilcoxon rank-sum test, multiple comparisons using Bonferroni's correction	13.385	13.9	0.11437572	0.99	
22	10*C* r_o point 3_	Non-Gaussian	Wilcoxon rank-sum test, multiple comparisons using Bonferroni's correction	17.683	18.53	0.0441126	0.926365	
23	10*C* r_o point 4_	Non-Gaussian	Wilcoxon rank-sum test, multiple comparisons using Bonferroni's correction	13.651	14.54	0.00450812	0.09467	
24	10*C* r_o point 5_	Non-Gaussian	Wilcoxon rank-sum test, multiple comparisons using Bonferroni's correction	17.135	18.34	0.00595367	0.125027	
25	10*C* r_o point 6_	Non-Gaussian	Wilcoxon rank-sum test, multiple comparisons using Bonferroni's correction	15.473	16.89	0.02489169	0.522726	
26	10*C* r_o point 7_	Non-Gaussian	Wilcoxon rank-sum test, multiple comparisons using Bonferroni's correction	12.647	14.16	0.00296237	0.06221	
27	10*C* r_o point 8_	Non-Gaussian	Wilcoxon rank-sum test, multiple comparisons using Bonferroni's correction	8.3481	9.956	0.00404024	0.084845	
28	10*C* r_o point 9_	Non-Gaussian	Wilcoxon rank-sum test, multiple comparisons using Bonferroni's correction	9.5921	11.33	0.00083891	0.017617	
29	10*C* r_o point 10_	Non-Gaussian	Wilcoxon rank-sum test, multiple comparisons using Bonferroni's correction	11.218	13.3	0.00049705	0.010438	
30	10*C* r_o point 11_	Non-Gaussian	Wilcoxon rank-sum test, multiple comparisons using Bonferroni's correction	10.879	13.25	0.00078763	0.01654	
31	10*C* r_o point 12_	Non-Gaussian	Wilcoxon rank-sum test, multiple comparisons using Bonferroni's correction	9.4174	11.85	4.167E-05	0.000875	

32	10*C* r_e point 1_	Non-Gaussian	Wilcoxon rank-sum test, multiple comparisons using Bonferroni's correction	6.9805	9.348	0.00142116	0.029844	
33	10*C* r_e point 2_	Non-Gaussian	Wilcoxon rank-sum test, multiple comparisons using Bonferroni's correction	8.6759	11.41	8.01E-05	0.001681	
34	10*C* r_e point 3_	Non-Gaussian	Wilcoxon rank-sum test, multiple comparisons using Bonferroni's correction	3.5517	5.998	2.64E-06	5.55E-05	
35	10*C* r_e point 4_	Non-Gaussian	Wilcoxon rank-sum test, multiple comparisons using Bonferroni'scorrection	8.3035	11.63	0.00058299	0.012243	
36	10*C* r_e point 5_	Non-Gaussian	Wilcoxon rank-sum test, multiple comparisons using Bonferroni's correction	2.7771	5.978	0.00077968	0.016373	
37	10*C* r_e point 6_	Non-Gaussian	Wilcoxon rank-sum test, multiple comparisons using Bonferroni's correction	10.731	14.87	0.00044245	0.009291	
38	10*C* r_e point 7_	Non-Gaussian	Wilcoxon rank-sum test, multiple comparisons using Bonferroni's correction	3.3448	6.996	0.00042872	0.009003	
39	10*C* r_e point 8_	Non-Gaussian	Wilcoxon rank-sum test, multiple comparisons using Bonferroni's correction	2.8413	5.523	0.0001793	0.003765	
40	10*C* r_e point 9_	Non-Gaussian	Wilcoxon rank-sum test, multiple comparisons using Bonferroni's correction	6.9591	10.57	0.00277197	0.058211	
41	10*C* r_e point 10_	Non-Gaussian	Wilcoxon rank-sum test, multiple comparisons using Bonferroni's correction	2.8669	5.47	0.00251034	0.052717	
42	11*C* r_o point 1_	Normal		1.5185	1.677	Baseline		
43	11*C* r_o point 2_	Normal	Unpaired parametric two-tailed *t* test with Bonferroni's correction	7.2033	7.494	0.00110981	0.023306	
44	11*C* r_o point 3_	Normal	Unpaired parametric two-tailed *t* test with Bonferroni's correction	3.2377	3.571	0.00013646	0.002866	
45	11*C* r_o point 4_	Normal	Unpaired parametric two-tailed *t* test with Bonferroni's correction	1.127	1.403	0.00013646	0.002866	
46	11*C* r_o point 5_	Normal	Unpaired parametric two-tailed *t* test with Bonferroni's correction	–0.101	0.198	0.00013646	0.002866	
47	11*C* r_o point 6_	Normal	Unpaired parametric two-tailed *t* test with Bonferroni's correction	6.6549	6.973	6.0108E-06	0.000126	
48	11*C* r_o point 7_	Normal	Unpaired parametric two-tailed *t* test with Bonferroni's correction	4.5553	4.844	3.4074E-06	7.16E-05	
49	11*C* r_o point 8_	Non-Gaussian	Wilcoxon rank-sum test, multiple comparisons using Bonferroni's correction	9.6173	9.987	6.0108E-06	0.000126	
50	11*C* r_o point 9_	Normal	Unpaired parametric two-tailed *t* test using Bonferroni's correction	12.422	12.77	2.3165E-06	4.86E-05	
51	11*C* r_o point 10_	Non-Gaussian	Wilcoxon rank-sum test, multiple comparisons using Bonferroni's correction	6.7661	7.099	2.3165E-06	4.86E-05	
52	11*C* r_o point 11_	Normal	Unpaired parametric two-tailed *t* test with Bonferroni's correction	7.7391	8.061	2.3165E-06	4.86E-05	
53	11*C* r_o point 12_	Normal	Unpaired parametric two-tailed *t* test with Bonferroni's correction	2.666	2.925	1.5022E-05	0.000315	
54	11*C* r_e point 1_	Normal	Unpaired parametric two-tailed *t* test with Bonferroni's correction	6.69	6.954	0.06043558	0.99	
55	11*C* r_e point 2_	Normal	Unpaired parametric two-tailed *t* test with Bonferroni's correction	0.3527	0.601	0.00218796	0.045947	
56	11*C* r_e point 3_	Normal	Unpaired parametric two-tailed *t* test with Bonferroni's correction	0.8799	1.111	0.00167504	0.035176	
57	11*C* r_e point 4_	Normal	Unpaired parametric two-tailed *t* test with Bonferroni's correction	2.401	2.666	0.02303299	0.483693	
58	11*C* r_e point 5_	Normal	Unpaired parametric two-tailed *t* test with Bonferroni's correction	1.2529	1.52	0.02075479	0.435851	
59	11*C* r_e point 6_	Normal	Unpaired parametric two-tailed *t* test with Bonferroni's correction	2.5986	2.875	0.00677512	0.142277	
60	11*C* r_e point 7_	Normal	Unpaired parametric two-tailed *t* test with Bonferroni's correction	2.166	2.394	0.06043558	0.99	
61	11*C* r_e point 8_	Normal	Unpaired parametric two-tailed *t* test with Bonferroni's correction	–1.413	–1.174	0.03444928	0.723435	
62	11*C* r_e point 9_	Normal	Unpaired parametric two-tailed *t* test with Bonferroni's correction	–2.322	–2.088	0.13825579	0.99	

63	11*C* r_e point 10_	Normal	Unpaired parametric two-tailed *t* test with Bonferroni's correction	3.104	3.334	0.6212314	0.99	
64	12*D* base	Normal	Unpaired parametric two-tailed *t* test	1.5767	21	1.10E-94		
65	12*D* PCC	Normal	Unpaired parametric two-tailed *t* test	2.5928	26.6			
66	12*E* base	Normal	Unpaired parametric two-tailed *t* test	–2.79	0.561	7.16E-68		
67	12*E* PCC	Normal	Unpaired parametric two-tailed *t* test	2.0998	5.664			
68	13*D* base	Normal	Unpaired parametric two-tailed *t* test	-5.117	39.83	2.20E-98		
69	13*D* PCC	Normal	Unpaired parametric two-tailed *t* test	–12.36	58.3			
70	13*E* base	Normal	Unpaired parametric two-tailed *t* test	–2.949	2.526	5.89E-85		
71	13*E* PCC	Normal	Unpaired parametric two-tailed *t* test	0.335	4.271			
72	14 sEPSC 1 Hz	Normal	Unpaired parametric two-tailed *t* test	–0.379	0.519	0.33030076	2.312105	
73	14 sEPSC 2 Hz	Normal	Unpaired parametric two-tailed *t* test	–0.622	0.625	compare		
74	14 sEPSC 3 Hz	Normal	Unpaired parametric two-tailed *t* test	–0.739	0.791	0.24094181	1.686593	
75	14 sEPSC 4–5 Hz	Normal	Unpaired parametric two-tailed *t* test	–0.779	0.845	0.00310931	0.021765	
76	14 sEPSC 6–7 Hz	Normal	Unpaired parametric two-tailed *t* test	–1.111	1.17	0.08681955	0.607737	
77	14 sEPSC 8–9 Hz	Normal	Unpaired parametric two-tailed *t* test	–1.517	1.7	0.20093123	1.406519	
78	14 sEPSC 10–11 Hz	Normal	Unpaired parametric two-tailed *t* test	–1.977	1.894	0.27526754	1.926873	
79	14 sEPSC 12 Hz	Normal	Unpaired parametric two-tailed *t* test	–2.176	2.161	0.32390836	2.267359	
80	14 sIPSC 1 Hz	Normal	Unpaired parametric two-tailed *t* test	–0.327	0.504	compare		
81	14 sIPSC 2 Hz	Normal	Unpaired parametric two-tailed *t* test	–0.541	0.661	0.12123449	0.848641	
82	14 sIPSC 3 Hz	Normal	Unpaired parametric two-tailed *t* test	–0.801	0.81	0.05927391	0.414917	
83	14 sIPSC 4–5 Hz	Normal	Unpaired parametric two-tailed *t* test	–0.992	1.081	0.00281511	0.019706	
84	14 sIPSC 6–7 Hz	Normal	Unpaired parametric two-tailed *t* test	–1.499	1.607	0.15648547	0.99	
85	14 sIPSC 8–9 Hz	Normal	Unpaired parametric two-tailed *t* test	–1.809	2.025	0.07761798	0.543326	
86	14 sIPSC 10–11 Hz	Normal	Unpaired parametric two-tailed *t* test	–2.376	2.553	0.02082827	0.145798	
87	14 sIPSC 12 Hz	Normal	Unpaired parametric two-tailed *t* test	–2.823	3	0.03252634	0.227684	
88	14–1 Hz EPSC vs IPSC	Normal	Unpaired parametric two-tailed *t* test			0.13411531		
89	14–2 Hz EPSC vs IPSC	Normal	Unpaired parametric two-tailed *t* test			0.33656269		
90	14–3 Hz EPSC vs IPSC	Normal	Unpaired parametric two-tailed *t* test			0.06173898		
91	14 –4 Hz EPSC vs IPSC	Normal	Unpaired parametric two-tailed *t* test			0.00257893		
92	14–5 Hz EPSC vs IPSC	Normal	Unpaired parametric two-tailed *t* test			0.00169706		
93	14–6 Hz EPSC vs IPSC	Normal	Unpaired parametric two-tailed *t* test			0.02608082		
94	14 –7 Hz EPSC vs IPSC	Normal	Unpaired parametric two-tailed *t* test			0.06611165		
95	14–8 Hz EPSC vs IPSC	Normal	Unpaired parametric two-tailed *t* test			0.15396775		
96	14–9 Hz EPSC vs IPSC	Normal	Unpaired parametric two-tailed *t* test			0.08192995		
97	14–10 Hz EPSC vs IPSC	Normal	Unpaired parametric two-tailed *t* test			0.09107412		
98	14–11 Hz EPSC vs IPSC	Normal	Unpaired parametric two-tailed *t* test			0.027338		
99	14–12 Hz EPSC vs IPSC	Normal	Unpaired parametric two-tailed *t* test			0.04712797		

## Results

### CFC classifies distinct SLE sub-states

First, we asked whether CFC features were able to classify SLE sub-states in the neocortex of mice. SLEs were induced using low-Mg^2+^ ACSF and the I_CFC_ matrices were computed on the resulting signal (Fig. 1*A*). The I_CFC_ values between 1- to 12-Hz low-frequency and 30- to 250-Hz high-frequency were reduced, normalized, then applied to a two-state HMM. To distinguish state S1, the inter-SLE state, and state S2, the SLE state, we computed the MPDs of the HMM. Spontaneous SLEs began 5.4 ± 0.8 min after the addition of low-Mg^2+^ ACSF, and recurred at 0.61 ± 0.13 SLEs per minute (for *N*s, see Table 2).

**Table 2.** Summary of duration distributions for SLEs and seizures

Model type (*n* = #	Shape parameter for γ-distributions [95% CIs]			Duration of specified intervals (mean ± SEM; *p < 0.001, Wilcoxon rank-sum test)		
SLEs or seizures/slices/subjects)	r_o_/r_o_’	r_s_/r_s_’	r_e_/r_e_’	r_o_/r_o_’	r_s_/r_s_’	r_e_/r_e_’
Low Mg^2+^ (*n* = 48/13/10)	1.8 [1.4 2.3]/1.01 [0.74 1.39]	1.6 [1.3 2.1]/1.84 [1.33 2.56]	1.7 [1.3 2.2]/0.72 [0.54 0.97]	29.6 ± 3.3 s/28.4 ± 3.4 s	25.2 ± 3.1 s/36.9 ± 3.0 s*	25.9 ± 2.3 s/5.04 ± 1.0 s*
4-AP (*n* = 28/7/7)	1.2 [0.8 2.3]/1.9 [1.2 3.2]	2.8 [1.6 4.9]/1.3 [0.81 9.8]	1.9 [1.1 3.2]/0.81 [0.51 1.3]	11.9 ± 1.6 s/15.1 ± 2.1 s	31.8 ± 4.1 s/33.4 ± 4.0 s	15.1 ± 2.0 s/12.7 ± 2.2 s
Human iEEG (*n* = 19/0/7)	0.47 [0.28 0.79]/0.94 [0.54 1.64]	1.1 [0.64 2.0]/1.98 [1.1 3.58]	3.3 [1.8 6.1]/0.73 [0.42 1.3]	27.5 ± 7.2 s/21.5 ± 6.0 s	28.6 ± 6.1 s/37.8 ± 6.1 s	21.2 ± 2.7 s/12.5 ± 2.9 s*

The width of the MPD is characterized by marginal errors in the HMM. We examined these errors at the onset and termination of the SLEs, and hypothesized that the SLE sub-states are the timeframe of these errors (Fig. 2*A*). To test this hypothesis, we correlated the duration of the errors with state S2 defined SLE duration and intensity (Fig. 2*B*,*C*). See Table 2 for the average duration of the onset interval r_o_, SLE state r_s_, and end interval r_e_. The sub-state intervals had positive moderate correlation**s** with the SLE state S2 duration, whereas the SLE state r_s_ had a positive strong correlation (Table 1, lines 1–6). In contrast, intensity of the SLE state S2 was moderately correlated with r_o_ and r_s_. Hence, SLE severity was correlated primarily with state r_s_; however, the duration of the SLE was correlated with all three intervals. These data supported the hypothesis that MPD values between 0 and 1 at the onset and termination of the events were transition sub-states into and out of the SLE state, r_s_. Hence, CFC between 1–12 and 30–250 Hz classified distinct SLE sub-states.

### SLE sub-states have deterministic underlying dynamical processes

To identify the type of dynamics underlying the SLE sub-states, we computed the γ-distribution fits of the distribution of the durations (Fig. 2*D*; Table 2). The shape parameter for the SLE state r_s_ was >1, suggesting that this classified state was statistically deterministic. Furthermore, both r_o_ and r_e_ duration histograms were fit to shape parameters >1, demonstrating that the putative transition sub-states were also statistically deterministic. To identify how these SLE sub-intervals compared to the interval between the end of the termination sub-state and the beginning of the onset transition sub-state (the inter-SLE interval), we repeated the γ fit distribution on the histogram of the durations of the inter-SLE interval. We observed the inter-SLE interval had a γ fit duration of 0.76 [0.54 1.1] (*n* = 46 inter-SLE intervals, 17 slices, 14 subjects). This suggested that the inter-SLE interval was random, whereas the sub-intervals of the SLE state S2 were statistically deterministic.

To confirm the relationship between our classified SLE state and that of an amplitude-based identification of onset and termination, we applied ROC based analysis (Fig. 3*D*). The threshold for defining the SLE state in S2 was varied to obtain sensitivity and specificity measurements for the ROC curve. We observed that our classification was not 100% perfect, but close to 1, as indicated by the AUC of the ROC < 1, and by using other tests of sensitivity and specificity (Table 4). On inspection of the traces we observed that field bursting activity that traditionally defines the onset and termination of seizures is sometimes observed before, or after, the classified onset and termination transition sub-states.

One possibility is that the CFC features that were used to classify the SLE sub-states were primarily associated with the wave form shape, and not due to the existence of true low to high-frequency CFC (Kramer et al., 2008; Aru et al., 2015; Jensen et al., 2016). To address this, we tested the HMM using reconstructed data that was obtained by the lower envelope of the original dataset (Fig. 3). This removed the high-powered HFOs while preserving the power of the LFOs (Fig. 3*C*). Then, we re-computed the CFC features from this dataset, and re-tested the HMM. To account for variability in wave form shape, data were reconstructed using 2 forms of interpolation, linear and cubic. Both linear and cubic interpolated datasets performed poorly when testing on the original low-Mg^2+^ trained HMM (Fig. 3*D*), as demonstrated by low ROC characteristics as compared to the original dataset (Table 4). These data suggest that the low-frequency wave form shape is not sufficient to classify the CFC-based of the SLE state.

**Figure 4.**
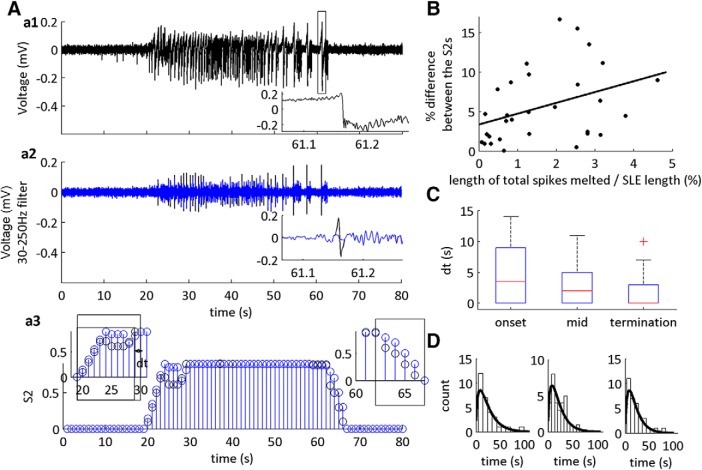
Large spikes do not affect the statistical distribution of SLE sub-states. ***A***, ***a1***, Example of a low-Mg^2+^ SLE. Inset is the expanded trace indicated by the box. It shows the occurrence of a large spike transient in the dataset. ***a2***, Example that has been filtered at 30–250 Hz. Black trace indicates original filtered signal, blue trace indicates filtered signal with minimized large spike transient. Inset is the expanded trace at time indicated by the box in ***a1***. ***a3***, MPD of classified state S2. Black is the original dataset and blue is the dataset with large spikes minimized. Insets are classified onset and termination transitions; dt is the difference between the classified transition sub-states. Boxes used to identify the onset and termination sub-states. ***B***, Correlation between the number of spikes removed from the datasets and the difference between the MPD of original S2 and the S2 with large spikes minimized (*r*^2^ = 0.1435, m = 1.37; b = 3.37; for Pearson correlation coefficient, see Table 1 line 7). ***C***, Boxplot showing the difference between the classified sub-state transitions as defined by the MPD of the original S2 and S2 with large spikes minimized. The whiskers are the data points that are not outliers. The outliers are plotted using the ‘+’ symbol. ***D***, Duration histograms of the onset, mid and termination intervals with the following γ-distribution shape parameter fits: 1.5 [1.0 2.4], mid 1.6 [1.0 2.5], termination 1.6 [1.0 2.6] (*n* = 30 SLEs, 13 slices, seven subjects).

To further identify the impact of spike shape on the transition classification, data were bandpass filtered at 30–250 Hz, and large amplitude spikes were smoothly excised using a spike melting algorithm (Fig. 4*A*,*a1*,*a2*; Zalay et al., 2009). The amplitude of this signal and the phase of the low-frequency signal was then used to generate a feature set. We trained each feature set on the original feature set, then tested it on the new feature set with spikes omitted (Fig. 4*Aa3*). We asked (1) how much does the number of excised spikes alter the MPD of the HMM? (2) what is the relative difference between the defined transition state durations with and without the large spiking transients? and (3) do these differences have an effect on the γ-distribution shape parameter fits of the duration histograms? We observed that there was a significant positive moderate correlation between the relative number of excised spikes and the overall difference in the MPDs of S2 (Fig. 4*B*; Table 1, line 7). This effect led to, on average, a 2-s difference between the classified sub-state durations, and no significant differences between the sub-states (Fig. 4*C*). Furthermore, the shape parameters of the γ-distribution fits of the SLE duration sub-states were >1 (Fig. 4*D*), as previously observed. These data demonstrate that the high amplitude transients impact the HMM classifier within a narrow margin of error, without altering the statistical distributions of the SLE sub-states.

### 4-Hz rhythm underlies statistical determinism of SLE transition sub-states

**Figure 5.**
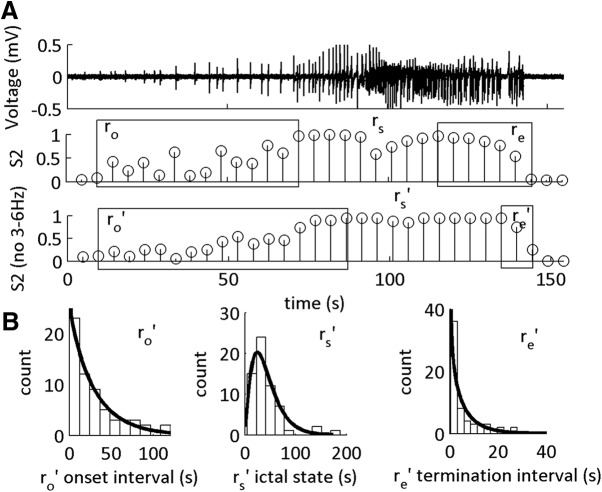
The statistical distributions of the SLE transition sub-states are altered on omission of 3–6 Hz from the CFC feature set. ***A***, Example of LFP (top) with MPDs of S2 tested using two feature sets. S2, The original feature set including 1–12 and 30–250 Hz. S2 (no 3–6 Hz), The feature set composed of 1–2.5 and 7–12 Hz. r_o_’, r_s_’, and r_e_’ are the updated durations of the SLE sub-states as defined by the MPD of S2 on omission of 3–6 Hz. ***B***, Histograms of the durations of r_o_’, r_s_’, and r_e_’ for all SLEs with their respective γ-distribution fits. For a summary of the shape parameters of the γ-distribution fits, see Table 2.

To identify what range of frequencies contained information about the deterministic properties of the SLE sub-intervals, we varied the frequency ranges of interest that were used to classify the sub-intervals of the SLE. Then we re-tested and re-trained the two-state HMM and re-computed the shape parameter fits of the γ-distributions obtained on their histograms (Table 3). As a representative example, we removed the 3- to 6-Hz frequency range from the original HMM feature set, and the model was re-trained and re-tested (Fig. 5). In this case, the onset and termination sub-states were Poisson distributed and random, respectively, as indicated by shape parameters ≤1, whereas the r_s_ state was statistically deterministic. Furthermore, we observed that the onset sub-state remained, on average, similar in duration to the original trained model. Yet, the termination sub-state was markedly reduced in the majority of tests. This was in parallel to a prolonged r_s_ state duration.

**Table 3.** Shape parameters for other combinations of training for low-Mg^2+^ SLEs

	Shape parameter for γ-distribution fit		
HMM range	onset (*p* = 0.05)	Mid (*p* = 0.6429)	End (**p = 0.0079)
1–12 Hz	1.8 [1.4 2.3]	1.6 [1.3 2.1]	1.7 [1.3 2.2]
1–4, 7–12 Hz	1.5 [1.0 2.4]	2.2 [1.4 3.4]	3.1 [1.9 5.0]
1–6 Hz	1.7[1.1 2.8]	2.2 [1.4 3.6]	2.9 [1.8 4.7]
2–6 Hz	1.2 [0.8 1.9]	2.4 [1.5 3.7]	1.6 [1.0 2.5]
3–5, 8–12 Hz	1.0 [0.6 1.5]	1.6 [1.0 2.6]	2.6 [1.6 4.1]
2–3, 5–10 Hz	0.73 [0.70 1.6]	2.7 [1.7 4.2]	0.75 [0.38 0.84]
1–2, 7–12 Hz	1.0 [0.74 1.39]	1.8 [1.33 2.56]	0.72 [0.54 0.97]
1–2, 5–12 Hz	0.93 [0.61 1.43]	3.0 [1.9 4.8]	0.55 [0.37 0.83]
1–3, 5–12 Hz	1.3 [0.82 1.9]	2.0 [1.3 3.2]	0.77 [0.5 1.0]
1–3 Hz	1.0 [0.9 2.1]	1.6 [1.4 3.3]	0.74 [0.50 1.11]

The *p* values indicated are for the shape parameters of the HMM ranges that contain 4 Hz to those that do not contain 4 Hz. The Wilcoxon rank-sum test was applied as this data were non-Gaussian.

Of 395 combinations of frequencies tested on our training set, 128 were successfully able to classify the SLE state, at varying degrees of accuracy. Training our dataset on a subset of ten cases, we preserved the statistical determinism of the middle r_s_ state. In all but one case where the 4 Hz was included in the model, the SLE transition sub-states had shape parameters >1. When this frequency band was not included in the model, the SLE transition sub-states had shape parameters ≤∼1. This suggested that information from the 4-Hz range was necessary to classify statistically deterministic durations of the SLE termination transition sub-state.

### Dynamics of seizure sub-states in humans can be distinguished using their underlying CFC features

**Figure 6.**
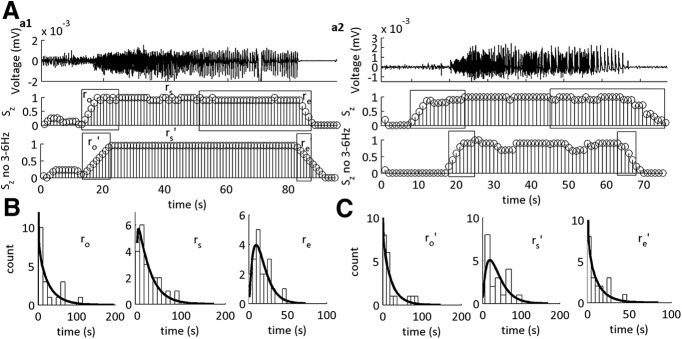
The statistical distribution of the human seizure termination sub-state is altered on omission of 3–6 Hz from the CFC feature set. ***A***, ***a1***, ***a2***, Two different examples of iEEG traces of human seizures (***a1***, patient 4; ***a2***, patient 2; top) with MPDs of S2 (S_Z_) tested using two different feature sets. Top, The original feature set including 1–12 and 30–250 Hz. Bottom, The feature set composed of 1–2 and 7–12 Hz. ***B***, Histograms of the durations of r_o_, r_s_, and r_e_ with their respective γ-distribution fits. ***C***, Histograms of the durations of r_o_’, r_s_’, and r_e_’ with their respective γ-distribution fits. For a summary of the shape parameters of the γ-distribution fits, see Table 2 (*n* = 19 seizures, seven patients).

To extend these findings to human seizures, we classified seizures that were recorded from patients before undergoing epilepsy surgery (Table 5). Because of interspecies variability, we re-trained the model to a human seizure trace, and then tested the remainder of the seizures obtained across these patients (Fig. 6*A*). We asked whether similar dynamical changes would result if we tested this model using the feature set with which we omitted the 3- to 6-Hz frequency range, as compared to that which included this range (Fig. 6*B*,*C*). When the SLE states were classified using the whole I_CFC_ feature set, the SLE and termination sub-states had shape parameter fits >1, whereas the SLE onset sub-state had a shape parameter < 1 (Fig. 6*B*; Table 2). When the 3- to 6-Hz range was omitted from the feature set, there was a marked difference in the shape parameters of termination distribution histogram (Fig. 6*C*; Table 2). The SLE termination transition sub-state duration shifted to a shape parameter fit < 1, suggesting information contained within the 3- to 6-Hz frequency range was correlated with the statistical determinism of the termination state.

### Testing in 4-AP-induced SLEs

**Figure 7.**
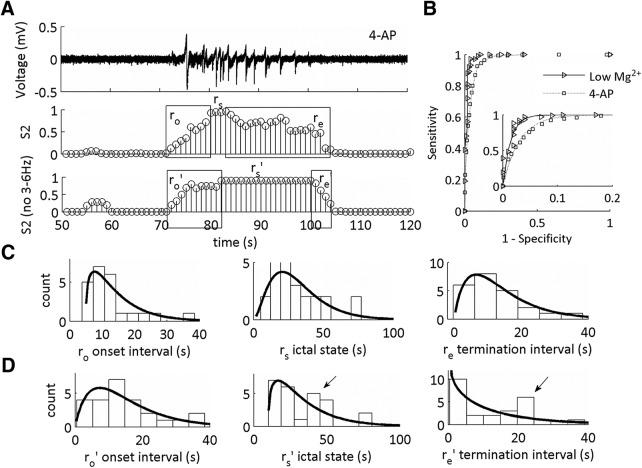
The statistical distribution of the 4-AP SLE termination sub-state is altered on omission of 3–6 Hz from the CFC features using low-Mg^2+^ SLEs for training the model. ***A***, Representative trace of a SLE induced on addition of 4-AP, 100 µM. Middle trace is the state S2 of the MPD of the HMM from the LFP trace using 1–12 and 30–250 Hz. Bottom trace is the state S2 of the MPD of the HMM from the LFP trace using 1–2.5 and 7–12 Hz features for model training. r_o_’, r_s_’, and r_e_’ are the updated durations of the SLE sub-states as defined by the MPD of S2 on omission of 3–6 Hz. ***B***, Receiving operating characteristics curves applied to both *in vitro* datasets as a result of training the HMM on one low-Mg^2+^ SLE trace and testing on both low-Mg^2+^ and 4-AP SLEs. Inset shows expanded exponential fits of a segment of the ROC curve. Refer to Table 2 for a complete characterization of the ROC curve parameters (low-Mg^2+^ SLEs: *n* = 46 SLEs, 14 slices, 10 subjects; 4-AP SLEs: *n* = 28 SLEs, seven slices, seven subjects). ***C***, Histograms of the durations of r_o_, r_s_, and r_e_ with their respective γ-distribution fits. ***D***, Histograms of the durations of r_o_’, r_s_’, and r_e_’ with their respective γ-distribution fits. See Table 2 for shape parameters of the γ-distribution fits and duration averages for each interval.

To further generalize the results, we classified SLEs that were induced using bath application of 4-AP, 100 μM. Application of 4-AP in neocortical slice preparations resulted in spontaneous SLEs that began 5.2 ± 0.8 min after the addition of the 4-AP solution, and then recurred at a rate of 0.42 ± 0.04 per minute (for *N*s, see Table 2). We asked whether the low-Mg^2+^ model was sufficient to train the network for classifying the 4-AP SLEs, the two-state HMM was trained using a feature set from the low-Mg^2+^ model, then tested on 4-AP (Fig. 7). The threshold for defining the SLE state in S2 was varied to obtain sensitivity and specificity measurements for the ROC curve. The 4-AP dataset was classified with high specificity and sensitivity (Fig. 7*B*; Table 4). Hence, the two-state HMM trained on low-Mg^2+^ SLEs classified the 4-AP-induced SLE state.

**Table 4.** Summary of statistics for ROC curves

	Low Mg^2+^	4-AP	Linear (reconstructed)	Cubic (reconstructed)
AUC	0.98	0.96	0.77	0.69
Specificity factor, ξ	0.014	0.031	0.23	0.31
Sensitivity	0.79	0.68	0.43	0.41
Specificity	0.98	0.96	0.86	0.83

**Table 5.** iEEG patient details

Number	Age	Sex	Duration (years)	MRI Findings	Engel class	Electrode placement	Pathology
1	41	M	14	Mesial temporal sclerosis	1	Right temporal	Mesial temporal sclerosis
2	22	M	9	Normal	1	Left temporal	Normal
3	31	M	22	Mesial temporal sclerosis	1	Right temporal	Mesial temporal sclerosis
4	26	F	4	Normal	1	Right frontal	Normal
5	35	F	20	Normal	1	Right temporal	Normal amygdal-hippocampal
6	36	F	22	Non-lesional	1	Left temporal occipital, parietal	Cortical micro-dysgenesis
7	21	M	n/a	Cortical dysplasia	1	Left dorsofrontal	Normal

We asked whether similar dynamical changes would result if we tested this model using the feature set with which we omitted the 3- to 6-Hz frequency range, encompassing the 4-Hz rhythm not necessary. When the SLE states were classified using the whole I_CFC_ feature set, the SLE intervals had a shape parameter fit >1 (Fig. 7*C*). When the 3- to 6-Hz range was omitted from the feature set, there was no difference in the duration the of SLE intervals as compared to the first feature set used (Table 2). However, there was a marked difference in the shape parameters of duration distributions histograms (Fig. 7*D*). The SLE termination transition sub-state duration had a shape parameter fit <1, suggesting information contained within this frequency range was correlated with the statistical distribution of the termination state. Yet, two distinctive peaks emerged in the r_s_ and r_e_ state, as indicated by the arrows in Figure 7*D*. This suggested that there were two separate dynamical activities present in this 4-AP model.

**Figure 8.**
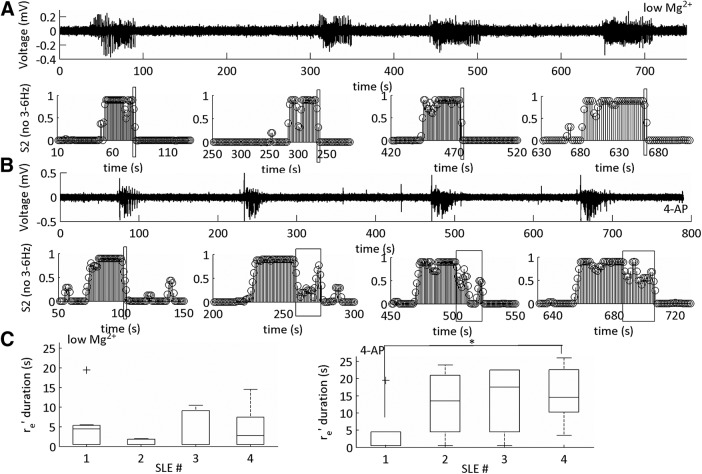
Temporal relationship of the SLE termination sub-state. ***A***, Representative trace of the low-Mg^2+^ SLE model with its respective MPD of the HMM. Boxes indicate SLE termination transition classification. ***B***, Representative trace of the 4-AP SLE model with its respective MPD of the HMM. Boxes indicate the SLE termination transition classification. ***C***, Boxplots showing the average duration of the termination transition of 4-AP and low-Mg^2+^ SLEs (*p < 0.05; 4-AP *n* = 24 SLEs, six slices, six subjects; low-Mg^2+^ *n* = 28 SLEs, seven slices, seven subjects; see Table 1, lines 8–15). The whiskers are the data points that are not outliers. The outliers are plotted using the ‘+’ symbol. For 4-AP, one subject was omitted from this calculation as only the first two SLE transition sub-states were classified accurately.

We theorized that the presence of two underlying dynamical distributions suggested a time-dependent change in the 4-AP SLE characteristics that was not present in the low-Mg^2+^-induced SLEs (Fig. 8). We grouped the first four SLEs along a given time interval within a single slice as numbers 1 through 4 for both datasets. For low-Mg^2+^, we observed that the r_e_’ sub-state was of short duration, and remained of short duration, over time (Fig. 8*C*, left). In the case of 4-AP, the r_e_’ sub-state duration was short for the first SLE relative to the 4^th^ SLE (Fig. 8*C*, right). These data suggested that the temporal dynamics of the 4-AP SLE model changed over the course of an experimental protocol, and, at least initially, a similar dynamical mechanism that contained information from the 4-Hz rhythm existed between both models.

### Identifying the 4-Hz HFO-coupled oscillation during the transition states in low-Mg^2+^ SLEs

**Figure 9.**
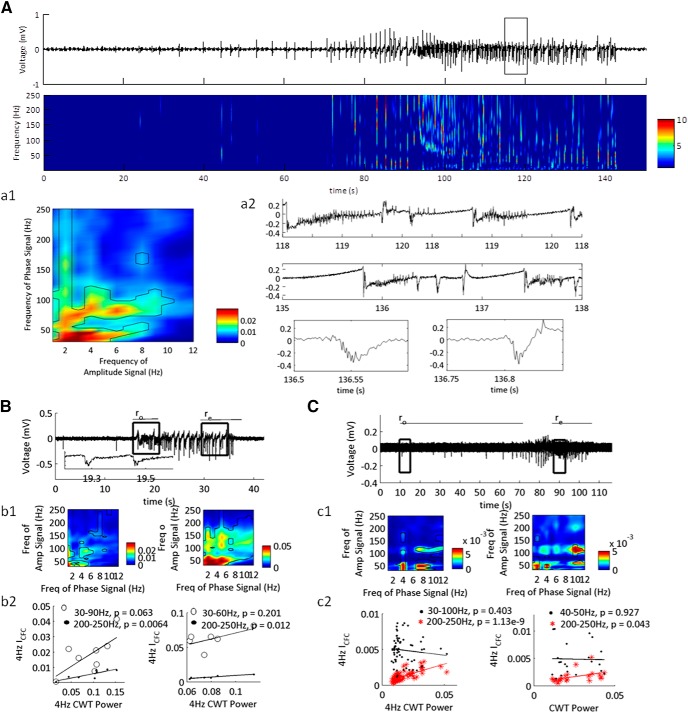
CFC coupled 4-Hz rhythm present during onset and termination phases of the SLE state. ***A–C***, Three examples LFP recordings of low-Mg^2+^-induced SLEs. Boxes indicate 5-s windows at which the CFC features were tested for significance. Inset in ***B*** is expanded trace from LFP segment showing HFOs present on LFOs. ***a1–c1***, CFC indices for the 5-s windows indicated by boxes. Regions of significance designated by black contour lines. ***a2***, Expanded traces from ***A*** demonstrating HFOs at specific phases of LFOs. ***b2***, ***c2***, Examples of correlation fits of the I_CFC_ and the cwt power of the 4-Hz oscillation during the r_o_ sub-states (left) and r_e_ sub-states (right) for representative examples (*p* values indicated for Pearson correlation coefficients; for average correlation coefficients, see Table 1, lines 16–19 *n* = 43 SLE, 14 slices, 10 subjects).

Given that the dynamics of the SLE sub-state transitions were dependent on the 4-Hz rhythm, we next wanted to confirm that this rhythm is a valid phase-amplitude CFC signal (Fig. 9). First, we inspected the unfiltered signal and observed that high-frequency activity of high amplitude occurred transiently on field bursting activity during the transition periods, some of which was oscillating at 4 Hz. Then, we applied surrogate analysis to distinguish the range of high-frequency signals that were significantly correlated with the 4-Hz rhythm. During a 5-s window at the beginning of the onset, and during a 5-s window at the beginning of termination, this range was variable, peaking between 30 and 100 Hz. Using this significant range for each SLE, we computed the correlation between the I_CFC_ and the power of the 4-Hz rhythm within the full transition states using 2-s windows to compute the CFC indices with 1-s overlap. If there was significant coupling, then this correlation would be either negatively correlated or not correlated with the 4-Hz signal power (Jensen et al., 2016). As a control, we also identified the correlation between the 4-Hz signal power and the average CFC indices from the 200- to 250-Hz range, which was not significantly coupled to the 4-Hz rhythm during the first 5 s of the onset transition and the first 5 s of the termination transition. For the 43 SLEs tested, 91% had a range of significant 4-Hz rhythm within the onset state, whereas at the termination state, 72% were significant. This is in comparison to the 200- to 250-Hz range with minimal significance during the onset and termination states (Table 1, lines 16–19). These data suggest that there is significant coupling between the 4-Hz oscillation at specific ranges of high-frequency rhythms, both at the onset and termination of the SLE state. During the termination sub-state, there is minimal coupling between the 4-Hz rhythm and the 200- to 250-Hz oscillation, as indicated by a *p* > 0.05. Because the average *p* value for the Pearson correlation coefficient during the onset transition was >0.05, likely the 4-Hz rhythm couples transiently to the 200- to 250-Hz range closer to the initiation of r_s._

### Excitatory and inhibitory current properties during the SLE transition sub-states

**Figure 10.**
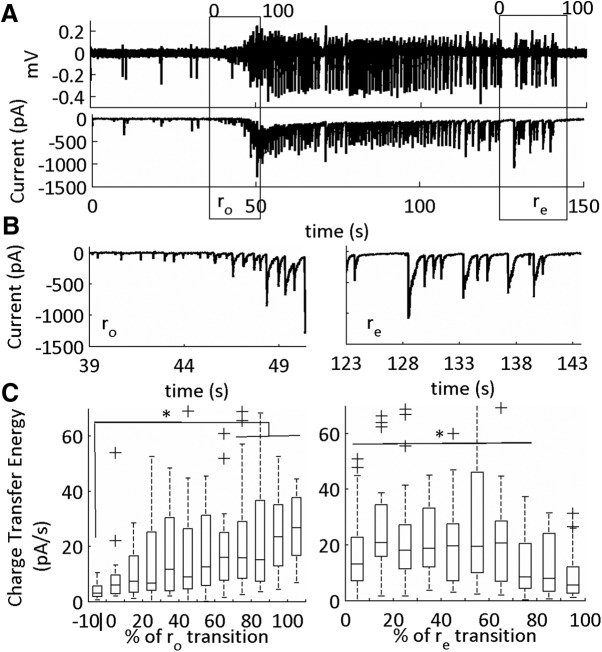
Excitatory currents (sEPSCs) in the onset (r_o_) and end (r_e_) sub-states of the SLE. ***A***, LFP (top) and intracellular voltage clamp trace (bottom) of a low-Mg^2+^-induced SLE. The SLE transition sub-states indicated by the boxes. The cells are held at –70 mV for sEPSC acquisition. The data were binned into 10 equal segments that on a scale of 0–100 corresponding to a percentage of the SLE transition sub-states. ***B***, Expanded traces of onset and termination sub-states from the representative example in ***A***. ***C***, Average charge transfer energy of the sEPSCs with horizontal lines at the top showing significance relative to pre-SLE baseline. The whiskers are the data points that are not outliers. The outliers are plotted using the ‘+’ symbol. Significance calculated relative to the 10% before the onset sub-state (*p < 0.05; 29 SLEs, 10 slices, nine subjects; Table 1, lines 20–41).

**Figure 11.**
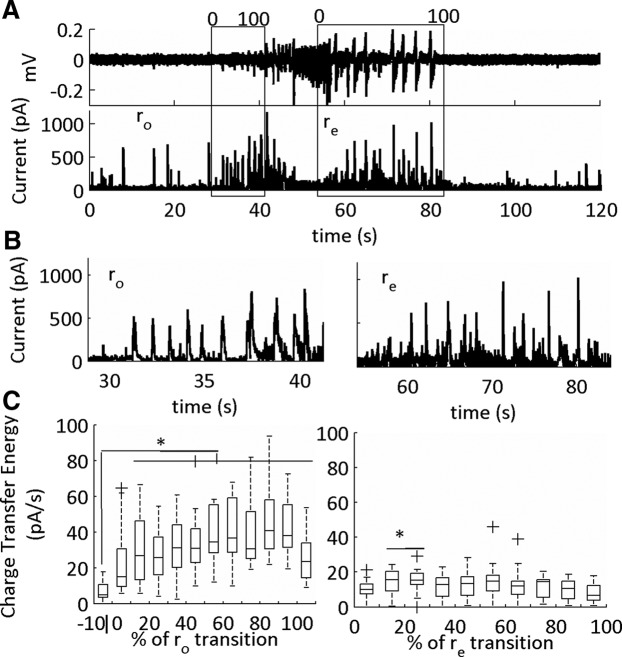
Inhibitory currents (sIPSCs) in the onset (r_o_) and end (r_e_) sub-states of the SLE. ***A***, LFP (top) and intracellular voltage clamp trace of a low-Mg^2+^-induced SLE. The SLE transition sub-states indicated by the boxes. The cell is held at 0 mV for sIPSCs acquisition. The data were binned into 10 equal segments that on a scale of 0–100 corresponding to a percentage of the SLE transition sub-state. ***B***, Expanded traces of onset and termination sub-states from representative example in ***A***. ***C***, Average charge transfer energy of the sIPSCs (*p < 0.05; 19 SLEs, six slices, five subjects, see Table 1, lines 42–63). The whiskers are the data points that are not outliers. The outliers are plotted using the ‘+’ symbol. Significance calculated relative to the 10% before the onset transition sub-state.

To track the temporal dynamics of excitatory and inhibitory currents during the SLE transition sub-states, pyramidal neurons from Layer II/III were whole-cell patched and voltage clamped at –70 mV (reversal potential for IPSCs) and 0 mV (reversal potential for EPSCs; Figs. 10, 11). Then, we computed the charge transfer energies for the SLE transition sub-states as previously defined using the HMM. Because each SLE had a different transition time, data were binned into 10 windows of analysis, representing a percentage of the full transition state. The onset transition sub-state also included 10% before the onset, which was used for significance, and 10% after the transition, which defined the beginning of the SLE state r_s_.

During the onset sub-state there was a large rise in the charge transfer energy of the sEPSCs. During the termination sub-state these currents remained elevated, then recovered to baseline levels at 80–90% of the termination sub-state (Fig. 10). In contrast, for the sIPSCs, there was a temporary rise in sIPSCs, which peaked, then dropped, at varying temporal windows during the transition interval (Fig. 11). Furthermore, the sIPSCs were at pre-SLE baseline levels during the SLE termination sub-state except for the initial 10–30% of the transition. Hence, large sEPSCs mark both the onset and termination SLE transitions, whereas sIPSCs dynamically peaked at varying intervals during both sub-states.

### Peak phase coherence between spontaneous excitatory and inhibitory currents and the low-frequency rhythm

**Figure 12.**
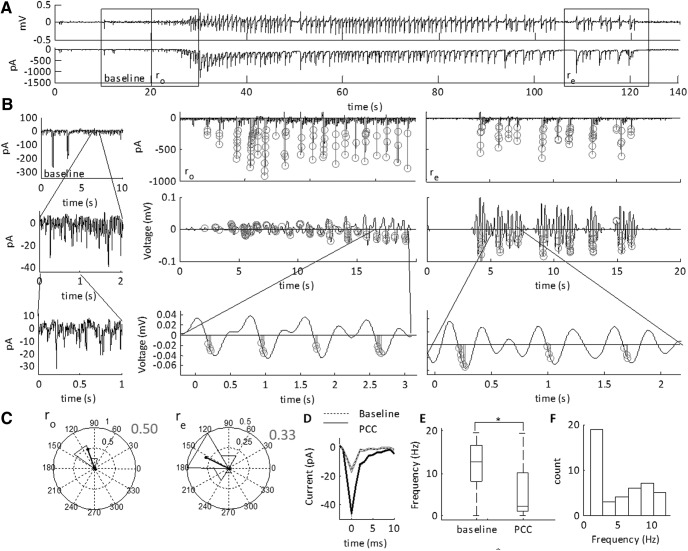
Phase coherent excitatory currents (PCCs) during the SLE transition sub-states. ***A***, LFP (top) and intracellular voltage clamp trace of a low-Mg^2+^-induced SLE. The SLE transition sub-states are indicated by the boxes. The cell is held at –70 mV for sEPSC acquisition. ***B***, Peak detection of the sEPSCs and the filtered LFP at 1–3 Hz during the onset (r_o_) and termination (r_e_) transition sub-states. Gray circles indicate detected sEPSCs. These are superimposed at the phase of the LFP oscillation at the time of detection. Thick black lines show average of the timing of the sEPSCs relative to the phase of the oscillation. ***C***, Circular histograms of phase at which peak of sEPSC was detected from example in ***B***demonstrating PLVs during the onset and termination phases indicated by the length of the black direction arrow. ***D***, Characterization of the PCCs as compared to baseline events (baseline). Baseline was chosen as 10 s just before the onset sub-state transition. The average amplitude of the PCC is 48 ± 2 pA (mean ± SEM), whereas the average amplitude of the baseline currents is 16 ± 2 pA (*p = 1.1E-94; 7402 baseline events, 1544 PCC events, 29 SLEs, 10 slices, nine subjects; see Table 1, lines 64–65). ***E***, Boxplots showing the median frequencies of sEPSCs with the 25th and 75th percentile ranges (*p < 0.001; see Table 1, lines 66–67). The whiskers are the data points that are not outliers. ***F***, Histogram of the frequency of maximal PLVs for the events showing moderate to high phase locking.

**Figure 13.**
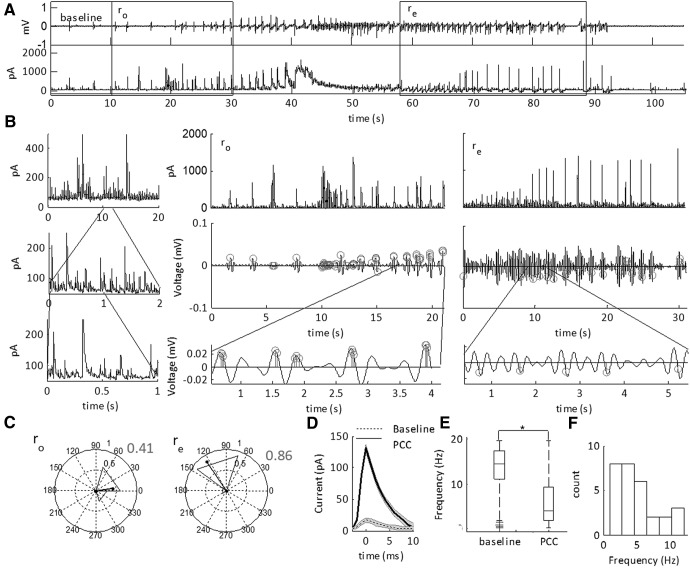
Phase coherent inhibitory currents during the SLE transition sub-states. ***A***, LFP (top) and intracellular voltage clamp trace of a low-Mg^2+^-induced SLE. The SLE state transition sub-states indicated by the boxes. The cell is held at 0 mV for sIPSC acquisition. ***B***, Peak detection of the sIPSCs and the filtered LFP at 1–3 Hz during the onset (r_o_) and termination (r_e_) state transition sub-states. Gray circles indicate detected sIPSCs. These are superimposed at the phase of the LFP oscillation at the time of detection. Thick black lines show the pattern of the timing of the sEPSCs relative to the phase of the oscillation. ***C***, Circular histograms of phase at which peak of sEPSC was detected from example in ***B***demonstrating PLV during onset and termination phases indicated by the length of the black direction arrow. Boxplot shows the frequency of maximal PLVs during SLE transition sub-states with the 25th and 75th percentile ranges (*n* = 19 SLEs, six slices, five subjects). ***D***, Average sIPSCs detected during the onset and termination sub-states. Baseline was chosen as 10 s just before the onset sub-state transition. The average amplitude is 128 ± 3 pA (mean ± SEM), the average amplitude of the baseline events is 15 ± 4 pA (*p* = 2.2E-98; 3155 baseline events, 606 PCC events, 19 SLEs, six slices, five subjects; Table 1, lines 68–67). ***E***, Boxplots showing the median frequencies of sIPSCs with the 25th and 75th percentile ranges (*p < 0.001; Table 1, lines 70–71). The whiskers are the data points that are not outliers. The outliers are plotted using the ‘+’ symbol. ***F***, Histogram of the maximal PLVs for the events showing moderate to high phase locking.

Next, we identified a population of currents that were coherent with the field rhythms (Figs. 12,13). We used a threshold measurement to identify peaks in sEPSCs and sIPSCs, and then varied the threshold until a PLV > 0.3 was observed (between 1 and 12 Hz). The threshold for detecting phase coherent currents (PCCs) was different for each SLE. For the sEPSCs (Fig. 12), during the onset and termination sub-states, 22/29 of the SLEs showed intermediate to high PLVs peaking at varying frequencies between 1 and 12 Hz for both onset and termination. In both sub-states, ∼70% of the cases were within the intermediate range, and 30% in the high range of PLVs We also used a Rayleigh test to test the cases for significance. In this case, during the onset, only 31% of the SLEs passed, which was consistent with those that had high PLVs Whereas, during the termination, 62% passed this test. Furthermore, there was no clear phase advancement or phase delay from onset to termination of the SLEs at the 4-Hz rhythm of interest. We hypothesized that the non-significant currents with moderate to high PLVs may, nonetheless, be integral to the dynamics of the transition sub-states.

We identified that the amplitude of these events was higher than those at baseline (Fig. 12*D*). Furthermore, the frequency of the currents at baseline was 13 Hz. This was also the same for the average frequency of all the spontaneous events during the transition sub-states. Yet, the PCCs had a median frequency of 2.3 Hz (Fig. 12*E*), and were spread across multiple frequency bands. This was further indicated by the histogram of maximal PLVs for all of the events showing moderate to high phase locking, which peaks at 1–2 and 9–10 Hz (Fig. 12*F*).

These findings were in contrast with the sIPSCs (Fig. 13). For both transition sub-states, 15/19 SLEs showed intermediate to high PLVs. From this, 63% of these were within the intermediate range, whereas 36% were within the high range. In contrast, during the termination, 54% of SLEs showed intermediate PLVs (0.36–0.44). The remainder were of low PLVs (<0.3). These were also phase coherent to a range of frequencies between 1 and 12 Hz. Using a Rayleigh test of significance, 73% of sIPSCs passed in the SLE onset, and 40% during the termination. Furthermore, the subset of common high PLVs (54%) had a phase advancement of 70 ± 11.2° phase angle difference from the onset to the termination. These results were not correlated with slice or subject number.

As with the sEPSCs, the amplitude of these events was higher than those at baseline (Fig. 13*D*). Furthermore, the frequency of the currents at baseline was ∼14 Hz. As with the sEPSCs, this was the same for the average frequency of all the spontaneous events during the transition sub-states. The sIPSC PCCs had an average frequency of 3.7 Hz (Fig. 13*E*), consistent with a correlation between the sIPSCs and the 4-Hz rhythm. This was further indicated by the histogram of maximal PLVs for all of the events showing moderate to high phase locking, which peaks at 1–4 and 11–12 Hz (Fig. 13*F*).

### Specificity of PCCs to SLE transition sub-states

To determine whether the PCCs were specific to the transition sub-states, we performed similar tests for identifying PCCs on the period 10 s before the SLE onset sub-state, and on the SLE state r_s_. During the inter-SLE period, only 12% sEPSC-SLE pairings showed significant and high PLVs, with a total of 5 detected events, and only 9% of the sIPSC-SLE pairings showed moderate PLVs with 19 total detected events. These results were similar during the ictal state, r_s_, where only 19% sEPSC-SLE pairings showed significantly moderate PLVs with a total of 35 detected events, whereas only 18% sIPSC-SLE pairings showed moderate PLVs with a total of 63 detected events. This suggested that the PCCs were specific to the onset and termination sub-state.

### sIPSCs show phase locking to the 4- to 5-Hz oscillation

**Figure 14.**
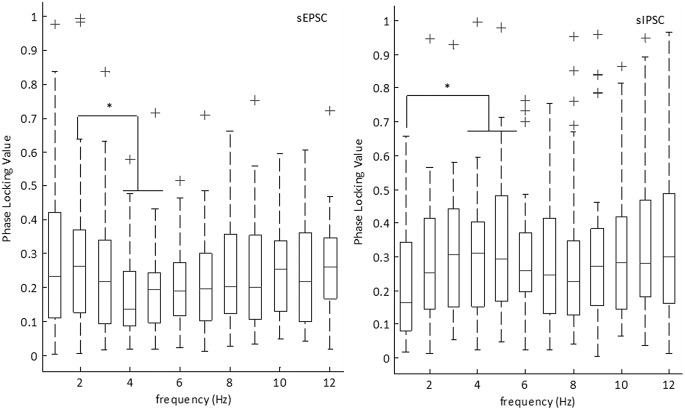
PLVs for sEPSCs and sIPSCs. Boxplot showing the median PLVs for sEPSC PCCs and sIPSC PCCs with their 25th and 75th percentiles. Whiskers indicate extreme data points that are not considered outliers. Outliers are indicated by the ‘+’ symbol. (*p < 0.05; Table 1, lines 72–99; sEPSCs *n* = 29 SLEs, 10 slices, nine subjects; sIPSCs *n* = 19 SLEs, six slices, five subjects).

To quantify the coherence between the intracellular currents and the low-frequency field oscillations, we calculated the frequency dependent correlation between the PLVs and the excitatory and inhibitory currents over the low-frequency range (Fig. 14). At the threshold for defining the PCCs, we examined the average PLVs at varying frequency bands over both onset and termination. For sEPSCs, we observed phase locking maximal at 2 Hz, consistent with the average frequency of the PCCs. For sIPSCs, we observed an elevated 4- to 5-Hz PLV, consistent with the average frequency of the sIPSCs. At 5 Hz, the median PLV breached the moderate phase locking threshold, suggesting sIPSCs, on average, moderately phase locked to 5 Hz. We then compared the PLVs between sEPSCs and sIPSCs (Table 1, lines 88–99). In this case, we observed elevated phase locking for both 4 and 5 Hz for sIPSCs as compared to sEPSCs. This suggests stronger phase locking between sIPSCs and the 4- to 5-Hz frequency range as compared to sEPSCs.

## Discussion

To the best of our knowledge, we are the first to demonstrate how specific CFC features demark statistical determinism of the SLE sub-states. We ask the following questions: what states do CFC features classify? Can these states be predicted? What is the underlying excitatory and inhibitory current bases for the predictability of the classified states?

### CFC classified SLE sub-states

We observe that CFC between the phase of 1–12 Hz and the amplitude of 30–250 Hz classify SLE state, and SLE onset and termination transition sub-states. A positive correlation between the SLE onset sub-state, and the SLE state, and SLE duration/intensity, suggests these intervals are distinct sub-states of the SLE. The termination state, which is comprised of bursting discharges, only positively correlates with the SLE duration. Field bursting activity that traditionally defines the onset and termination of seizures (Dreier and Heinemann, 1991; Boido et al., 2014; Ellender et al., 2014), is sometimes observed before the classified onset sub-state and after the classified termination sub-state. Field bursting activity is composed of low-frequency and high-amplitude events; however, to make the feature set, we used only information from the phase of the LFOs. An HMM is known to capture essential patterns within features, with the assumption that each pattern performs a distinct role in the network (Rabiner and Juang, 1986); hence, the output is highly dependent on our choice of features. Using the CFC features, with only low-frequency phase information to run the HMM, classifies sub-intervals of the SLEs that were not previously observed.

### Can seizure sub-states be predicted?

The CFC chosen for the HMM classifies three distinct SLE intervals that are statistically deterministic, *in vitro*. The histogram of the duration of the three SLE sub-intervals had a shape parameter >1, suggesting that both the onset and termination of SLEs are statistically deterministic. This is in line with previous results demonstrating that a deterministic process underlies the seizure termination state; hence, it can be predicted (Bauer et al., 2017). This is also consistent across both low-Mg^2+^ and 4-AP SLE models, and in human iEEG seizures. Furthermore, like with low-Mg^2+^-induced hippocampal SLEs (Suffczynski et al., 2006), the SLE onset is statistically deterministic, as indicated by a shape parameter >1. Yet, the previous work suggested that this is an exception in the low-Mg^2+^ model, as the interseizure intervals from human seizures have a shape parameter <1, and therefore are randomly occurring and unpredictable. These previous results computed the duration of the full interseizure interval from both human and animal models. We also obtained a shape parameter <1 for the human iEEG onset sub-state, identifying this sub-state as random in humans. As our results focused on the seizure state and its respective sub-states, future studies may examine interictal human iEEG durations to determine if a predictable interictal period will emerge on removal of the unpredictable seizure onset sub-state.

The random occurrence of the SLE state is also observable in our model, *in vitro,* if we remove the transition sub-states from the computation of the inter-SLE interval. These results are also consistent with the analysis done in *in vivo* data (Colic et al., 2013), and further confirmed in a computational model (Grigorovsky and Bardakjian, 2018). Hence, the beginning of the onset transition sub-state may emerge randomly; however, the transition into the SLE state, r_s_ emerges predictably, *in vitro*. Therefore, CFC features classify predictable sub-intervals of the SLEs that were not previously observed.

We consider alternatives when making the assertion of predictability. First, a γ-distribution shape parameter >1 can also be the addition of multiple random variables that are exponentially distributed with the same averages (Cox and Lewis, 1978). Yet, the consistency across models and species, suggests that underlying dynamical mechanisms are at work. Second, we assume that statistical determinism implies predictability. Third, this predictability could be an artifact of the HMM, as it has previously been demonstrated that the HMM can predict human seizure onset using the iEEG signal directly (Wong et al., 2007). Yet, the transition to seizures can be detected in advance of the clinical onset using CFC and a different classification approach, suggesting the seizure onset is predictable and this is not an artifact of the HMM (Jacobs et al., 2018). Changes in CFC features have been demonstrated as biomarkers of hyperexcitable brain states (Guirgis et al., 2015; Weiss et al., 2016; Grigorovsky and Bardakjian, 2018; Samiee et al., 2018). Therefore, we have demonstrated that seizure transition probability can be monitored by changes in the underlying network excitability (Kalitzin et al., 2005). As such, we have found a novel SLE interval, *in vitro*, that has predictable underlying dynamics independent of the inter-SLE interval.

### The 4-Hz phase underlies SLE transition sub-state predictability

Statistical determinism of the low-Mg^2+^ SLE onset and termination sub-states is observable with information contained in the 4 Hz frequency band. We observe a 4 Hz signal which, when omitted, results in ∼1 (confidence interval (CI): [0.81 1.9]) for the onset, and <1 (CI: [0.5 1]) for the termination. Although a slightly larger interval that contains the 3 Hz leads to random underlying dynamics <1 Hz (CI: [0.4 0.8]) for seizure termination, the striking shift to a near-Poisson distribution on omission of the 4 Hz is of interest, as it provides information about a network that shifts from predictable to unpredictable.

Previous work has demonstrated that there are multiple sub-states within the SLE and during pre-SLE state (Guirgis et al., 2014). The sub-states of the SLE were previously classified using 4–8 Hz, which contained sufficient network information to classify three sub-states of the SLE state in the hippocampus, which is associated with physiologic θ-γ coupling in the neocortex (Canolty et al., 2006). The transition sub-state histogram distributions are deterministic only when information contained within the 4-Hz range is added to the HMM. Hence, we need only this range to capture the information of statistical determinism of the transition sub-states, overlapping with these previous results. In contrast, for the 4-AP SLE model, omitting this range only affects the mid-SLE state and the termination transition sub-state in 4-AP, rather than the onset state. Furthermore, omitting 3- to 6-Hz range from human SLEs only effects the termination sub-state. This suggests the underlying dynamical mechanisms of SLE onset in the 4-AP model, and in human seizures, are not dependent on 4 Hz. Hence, the 4-Hz range most likely underlies the statistical determinism of seizure termination across models.

This is also confirmed when we observed that the removal of a range which contains the 4-Hz oscillation results in a large reduction in the duration of the classified termination sub-state, with only minor differences in the onset sub-state duration, yet a high proportion of SLEs have PCCs that are phase locked to this rhythm during the onset. We ask why the 4-Hz rhythm only has a major effect on the duration of the termination. It has previously been observed that a 400- to 800-Hz signal emerges during the onset transition of low-Mg^2+^ SLEs in the hippocampus (Lasztóczi et al., 2004). These findings were further confirmed in a similar SLE model (Khosravani et al., 2005). Also, in the cortex, fast ripples >250 Hz have been implicated in the initiation of seizures (Bragin et al., 1999; Jacobs et al., 2008). Therefore, this 4-Hz oscillation may have effects on the onset sub-state, but coupled with a higher frequency range that was not added to the model features; hence, not sensitive in the classifier.

This higher frequency activity in the fast ripple range is likely a summation of pyramidal cell burst spiking in the hippocampus (Dzhala and Staley, 2004). The neocortex may have a similar underlying mechanism. This is observed in our model as a large rise in charge transfer energy of sEPSCs during the onset phase. But these are coherent with a range of frequencies 1–12 Hz within the onset sub-state, clustering at 1–2 and 9–10 Hz. This suggests that a large proportion of the onset duration transition is correlated with the activity of pyramidal neuron spiking, and only a small proportion shifts the underlying dynamics of the onset.

### Excitatory and inhibitory current networks in SLE transition sub-states

The correlation between synaptic currents and the onset of low-Mg^2+^ SLE model has been identified previously (Traub et al., 1994). The faster onset time that is a possibility is unlikely related to the CFC based transitions observed because we observe a consistent slow rise of excitatory currents during the classified transition sub-state, and slow rise, then fall, of inhibitory currents. Furthermore, this pattern parallels multiple previous reports *in vitro* and *in vivo* (Kawaguchi, 2001; Timofeev and Steriade, 2004; Trevelyan et al., 2006). However, the traditionally defined onset that consists of the largest burst of inhibition synchronous with excitation previously observed (Trevelyan et al., 2006), did not consistently correlate with r_s_ SLE onset. Furthermore, the PCCs we observe that are specific to these transition sub-states, and are of larger amplitude than baseline, are related to bursts of inhibition and excitation temporarily coherent with the field activity. This is also confirmed by the observation that these PCCs are not following the average spontaneous event frequency during the transition sub-states and pre-SLE baseline (∼13 Hz) but are following a much lower frequency (2–4 Hz).

### Phase locking of the GABAergic network to the 4-Hz oscillation

Because of the higher phase locking of the sIPSCs around 4 Hz, and scattered phase locking of the sEPSCs across multiple frequencies 1–12 Hz, we observed significantly elevated PLVs of sIPSCs compared to sEPSCs in the 4-Hz frequency bands. Hence, we suggest that the sIPSCs make a stronger contribution to the 4-Hz rhythm than the sEPSCs. It is also in line with previous reports of phase locking of action potentials during the SLE onset transition in the low-Mg^2+^ model (Quilichini et al., 2012). Our results validate these previous findings in this older animal model in the neocortex. Differences in the overall phase locking strength could also be the timing and amount of excitatory and inhibitory connections received by our recorded pyramidal neurons (Huh et al., 2016). Also, it could be the number of detected events. Yet, we only observed PLVs significant and above 0.3 within distinct frequency bands at onset and termination, and we used a large number of events for each case. Therefore, our results are likely due to the underlying network activity rather than the number of events.

Given that we did not block sodium conductance it is likely that these PCCs are, in part, neuronal action potentials (Buzsáki et al., 2012). Our examination of currents rather than action potentials limited the peak detection algorithm to the refractoriness of the currents, which we set at a maximum of 20 Hz. This suggests that although there may be higher frequencies of PCCs (in the range of HFO amplitude detection) we cannot test this using our current approach. Also, given we used a threshold method for detection of peak currents, there may be PCCs during the SLE state that are not distinguishable using our approach (Ziburkus et al., 2006; Cotic et al., 2011). Nonetheless, another lab has observed that HFOs maximally coupling to the 4-Hz oscillation reflect the summation of IPSPs in pyramidal neurons (Jefferys et al., 2012). The summative effects may be why the PCCs we observe are of much higher amplitude than the baseline events. Another lab has observed phase locking of action potentials occurs during multiple phases of the SLE state (Lévesque et al., 2016). Given the specificity of the PCCs to the SLE transition sub-states, action potential spiking during the various phases of the SLE state does not reflect the postsynaptic neuronal response.

It has been previously observed that interneurons which fired preferentially at θ (4–10 Hz) increase their frequency during the transition to the ictal event (Karunakaran et al., 2016); whereas there is a concomitant slowing (or static activation) of pyramidal neuron action potentials (Grasse et al., 2013; Lévesque et al., 2016). Yet, we observed that the postsynaptic neuron is receiving a static population of both high amplitude excitatory and inhibitory currents during the transition sub-states. This suggests that a non-action potential dependent mechanism synchronizes the inputs from the population of excitatory and inhibitory neurons during the transitions of SLEs.

Unlike previous results that focused on either SLE onset or termination, we report the presence of PCCs during multiple phases of the SLE, therefore can compare the phase relationships between the state transitions. Specifically, when focusing on the 4-Hz frequency to calculate instantaneous phase at which the PCCs occur, we observe a phase advancement for the sIPSC PCCs in the termination sub-state relative to the onset sub-state, whereas this observation was not consistent with the excitatory currents. This further validates our hypothesis that sIPSCs play a dominant role in the 4-Hz network. Others have demonstrated that inhibitory cells tend to fire action potentials during the trough of the 5- to 15-Hz oscillation, suggesting the currents may exhibit a particular phase preference (Lévesque et al., 2016). Yet, we did not observe a consistent phase relationship between SLEs during the onset or the termination of the SLEs. These previous results were reliant on observations made from bandpass filtered signals, whereas our instantaneous phase from the cwt, may have resulted in different conclusions. This may also be due to intrinsic cellular mechanisms affecting the timing of the detected peaks, such as dynamic changes in ionic currents, or cell swelling. Yet, it may also suggest that the inhibitory current population active at termination is different from the onset (Ellender et al., 2014; Wen et al., 2015).

This work supports the concept that GABAergic cells are able to synchronize local neuronal networks (Chagnac-Amitai and Connors, 1989; Cobb et al., 1995; Quilichini et al., 2012; Chang et al., 2018). We observed that inhibition predominantly phase locks to the 4-Hz oscillation. We observe this in both the frequency of the PCCs at ∼4 Hz, and the PLV of these currents significantly higher at the 4 Hz for inhibition relative to excitation. The presence of a 4-Hz oscillation has been previously observed as part of the θ rhythm in the neocortex (Siapas et al., 2005; Sirota et al., 2008; Fujisawa and Buzsáki, 2011). But, the 4 Hz on its own has been demonstrated as a task-specific rhythm phase locked with GABAergic networks (Fujisawa and Buzsáki, 2011). This previous study observed a larger fraction of interneurons than pyramidal neurons phase locked to the 4-Hz oscillation in the pre-frontal cortex. We observe a similar phase locking bias in the somatosensory cortex under epileptic conditions. In this θ range, interneurons have been observed to synchronize network oscillations via gap junctions (Galarreta and Hestrin, 1999; Gibson et al., 1999; Beierlein et al., 2000; Tamás et al., 2000; Blatow et al., 2003). Hence, the synergistic action of chemical synapses between interneurons and pyramidal neurons and electrical synapses between interneurons may not only synchronize the neuronal populations in the context of maintaining a physiologic 4-Hz rhythm; it may further play a role in shaping the dynamics of SLE sub-intervals.

### Future directions

Our results uncover a population of large amplitude currents, both excitatory and inhibitory, that show peak phase-coherence with LFOs emerging during the transition sub-states of SLEs. Our observations lead to the following conclusions: There is higher phase locking of inhibition to the 4-Hz oscillation. The omission of the 4-Hz oscillation leads to Poisson distributed SLE transition sub-states, and the Poisson distribution follows that of the naturally occurring action potential firing frequency (Brunel, 2000). This suggests that inhibition maintains the predictability of the SLE transition sub-states. Future studies may consider targeting the coherent inhibitory events for direct modulation of the seizure termination phase. This work merges the underlying cellular mechanisms to fast and slow rhythms in the neural-glial network of the brain. In doing so, it can help unravel mechanism behind the activation of the local network during seizures and will guide future studies in the mechanisms underlying endogenous seizure transition dynamics.

## Synthesis

Reviewing Editor: Liset Menendez de la Prida, Instituto Cajal CSIC Decisions are customarily a result of the Reviewing Editor and the peer reviewers coming together and discussing their recommendations until a consensus is reached. When revisions are invited, a fact-based synthesis statement explaining their decision and outlining what is needed to prepare a revision will be listed below. The following reviewer(s) agreed to reveal their identity: Michael Cohen.

Your ms was reviewed by two independent referees and myself and we all agree there is potential value in your data. However, we also agreed there is a major methodological concern regarding the possibility of spurious CFC due to artifacts of sharp transients. There was also some discussion on how general/relevant your observation is to the human context.

After some discussion we decided to grant you with the possibility to address the several concerns. Here is a list of those issues:

Major comments:

1- CFC is known to be highly sensitive to spikes or other sharp transients in the data. There are many publications about this confound in the literature, including from groups of Tort, Cohen, Voytek, and Singer. In other words, a sharp transient, or even a non-sinusoidal waveform shape, can cause spurious CFC. This basically results from the Fourier transform of an edge, which causes ripples at higher frequencies and phase “reset” at lower frequencies. We feel this appears to be the case here. Thus, we feel you may have simply identified brief large-derivative time periods as being predictive of epileptic activity. We therefore ask you to implement some control analyses to determine whether there is any true CFC in the data, or whether the results are driven by an “artifact”.

We suggest a careful read of the literature for some inspiration on how to address the potential confound (e.g., Aru et al 2014; Jensen et al 2016; Kramer, Tort, Kopell 2008 [most relevant]). We also suggest two control analyses: (1) redo the analyses but excluding any data periods that contain particularly large spikes; (2) simulate data without cross-frequency coupling but which otherwise looks morphologically similar to the empirical traces, and then apply the same analyses.

2- Your findings would be of broader interest if they were transferrable to human epilepsy. Intracranial recordings of human neocortical seizures are readily available online, e.g., in the “European database on epilepsy”, and epilepsy centers at Harvard or Columbia are using Utah arrays to record LFP in these seizures. There may be also the possibility of you having acces to in vitro data from human slices already published by the lab. If you could generalize your observation to the human epileptic context we feel the work will gain impact.

Other comments:

(1) l.39: Phase amplitude cross frequency coupling was first described in humans by Mormann et al., 2005 (Hippocampus 15:890). This study should be cited instead of (or in addition to) Cohen et al.

(2) ll.64-66: “Therefore, simultaneous macroscopic and microscopic measurements provide a substantial advancement in identifying the underlying cellular mechanisms of seizure sub-states.” The authors use LFP recordings, which no epileptologist would consider macroscopic. Maybe use extracellular vs. intracellular instead.

(3) ll.173-175: “The process was repeated to the point where either the difference between log likelihood values of successive iterations dropped

below 10-5 or 100 iterations took place.” How often did each of these outcomes occur?

(4) l.184: The different phases r_o, r_s, and r_e are mentioned here for the first time, but not explained.

(5) ll.189-191: “To compare to the 'gold standard; approach to defining seizure onset and termination, these were identified via visual inspection of the LFP signal. The SLE was defined as between the times where the LFP signal rose to a 3x the baseline signal.” So which of the two was it? Visual inspection or 3x the baseline?

(6) l.272: “[...] we varied the frequency ranges of interest that were used to classify the sub-intervals of the SLE.” How many ranges did you try out? Only the ones listed in Tab. 3?

(7) ll.373-374: “[...] specifically, this amplitude was not dependent on their timing during the transition sub-states, nor on the sub-state (figure 10d).” This is also stated in the captions of Figs. 9 and 10, but I cannot seem to deduce it from the figure. Please explain.

(8) ll.452-458: “results in a ~1Hz [...], and &lt; 1Hz”. I'm assuming this relates to the shape parameter alpha, in which case the unit “Hz” does not make any sense.

(9) ll. 504 and 552: Both these lines contain the section heading “Phase locking of the GABAergic network to the 4Hz oscillation”. Please decide where you want to put it.

(10) l.507: “compared to sIPSCs in the 4Hz frequency bands”. Shouldn't this be “compared to sEPSCs in the 4Hz frequency bands”?

(11) l.536: The section heading “PCC advancement of IPSCs” should read “Phase advancement of IPSCs”

(12) ll.764-766: Figure 2C: Why is the correlation for r_e weak and that for r_o moderate? They both look the same.

(13) ll.797-799: Figures 6A&B: “Boxes indicate the SLE termination transition classification.” What boxes?

(14) l.828: “baseline events” Please define baseline.

(15) Table 2: For “Normal” (i.e., Gaussian) “Data Structure”, the authors sometimes use a t-test, sometimes a Wilcoxon rank sum test (e.g. Line # 38, 40). Please explain.

Typos.

-l.5: “remains” should be “remain”.

-l.100: “Muliclamp” should be “Multiclamp”.

-l.189: “the 'gold standard; approach” should be “the 'gold standard' approach”.

-l.278: “the onset state sub-state” should be “the onset sub-state”.

-l.467: “the 4-AP SLE model” should be “for the 4-AP SLE model,”.

-l.523: “reflects” should be “reflect”.

-ll.797,798: “it's” should be “its”.

-Throughout Table 2: “Boneferroni” should be “Bonferroni”.

-eq. 4 is missing a parenthesis in the numerator.
